# *Haliskia peterseni*, a new anhanguerian pterosaur from the late Early Cretaceous of Australia

**DOI:** 10.1038/s41598-024-60889-8

**Published:** 2024-06-12

**Authors:** Adele H. Pentland, Stephen F. Poropat, Ruairidh J. Duncan, Alexander W. A. Kellner, Renan A. M. Bantim, Joseph J. Bevitt, Alan M. Tait, Kliti Grice

**Affiliations:** 1 Western Australian Organic & Isotope Geochemistry Centre, School of Earth and Planetary Science, Curtin University, Bentley, Western Australia 6102 Australia; 2 Australian Age of Dinosaurs Museum of Natural History, The Jump-Up, Winton, Queensland 4735 Australia; 3 School of Biological Sciences, Faculty of Science, Monash University, Clayton, Victoria 3800 Australia; 4 Museums Victoria Research Institute, GPO Box 666, Melbourne, Victoria 3001 Australia; 5 Laboratório de Sistemática e Tafonomia de Vertebrados Fósseis, Setor de Paleovertebrados, Departamento de Geologia e Paleontologia, Museu Nacional, Universidade Federal do Rio de Janeiro, Rio de Janeiro, Brazil; 6 Museu de Paleontologia Plácido Cidade Nuvens, Universidade Regional do Cariri, Santana do Cariri, Crato, Ceará Brazil; 7 Australian Centre for Neutron Scattering, Australian Nuclear Science and Technology Organisation, Sydney, New South Wales 2234 Australia

**Keywords:** Palaeontology, Zoology

## Abstract

Pterosaur remains have been reported from every continent; however, pterosaur skeletons remain rare. A new pterosaur is presented here, *Haliskia peterseni* gen. et sp. nov., constituting the most complete specimen from Australia from the upper Albian Toolebuc Formation of the Eromanga Basin (Queensland, Australia). A combination of features, including the presence of a premaxillary crest and curved teeth, and the morphology of the scapulocoracoid, support its referral to Anhangueria. *Haliskia* can be distinguished from all other anhanguerian pterosaurs based on two dental characters: the 4th and 5th tooth pairs are smaller than the 3rd and 6th, and the 2nd and 5th alveoli are smaller than 3–4 and 6–8. Moreover, the hyoid is 70% the total length of the mandible. The phylogenetic analyses presented here place *Haliskia* within Anhangueria. In one analysis, *Haliskia* and *Ferrodraco* are resolved as sister taxa, with *Tropeognathus mesembrinus* sister to that clade. The other resolves *Haliskia*, *Mythunga* and *Ferrodraco* in a polytomy within Tropeognathinae. The new Australian pterosaur attests to the success of Anhangueria during the latest Early Cretaceous and suggests that the Australian forms were more taxonomically diverse and palaeobiogeographically complex than previously recognized.

## Introduction

Fossils of pterosaurs are rare in eastern Gondwana, in stark contrast to their relative abundance and diversity in western Gondwana^1–6^. Consequently, understanding the evolutionary history of pterosaurs in eastern Gondwana remains a challenge: resolving the phylogenetic affinities of several pterodactyloid clades has been impeded by the dearth of material from Australia, New Zealand, Antarctica, Indo-Pakistan, and Madagascar^7,8^. Almost all specimens reported from eastern Gondwana consist of isolated and fragmentary remains, with the exception of ‘*Campylognathoides indicus*’^9,10^ and *Ferrodraco lentoni*^11,12^. ‘*Campylognathoides indicus’* is a problematic taxon^13^, as the pterosaurian affinities of the referred cranial material have been questioned^14^. This is further compounded by the lack of anatomical overlap between the holotype and referred specimens^14,15^, and the fact that the phylogenetic position of this taxon has not been rigorously assessed. By contrast, the holotype of *Ferrodraco lentoni* (represented by cranial, axial and appendicular elements), has been repeatedly resolved as a member of Anhangueria^12,16^.

Although most of the Australian pterosaur material derives from the Toolebuc Formation of Queensland^17–24^, all specimens reported from this unit to date are isolated and fragmentary. These include indeterminate anhanguerians^17,18, 20, 21, 24^, as well as three of the four named Australian pterosaur taxa: the anhanguerians *Mythunga camara*^19,25^ and *Thapunngaka shawi*^23,26^, and the targaryendraconian *Aussiedraco molnari*^22,27^. Phylogenetic analyses have clarified the affinities of all three named taxa, with a close relationship between *Mythunga* and *Ferrodraco* supported in several phylogenetic analyses^11,12^. These are sometimes resolved in a polytomy with either *Tropeognathus mesembrinus*^12,16^ or *Thapunngaka shawi*^23,26^, and the latter result led to the erection of the clade Mythungini^26^. Despite these advances, the precise phylogenetic position of the Australian anhanguerians remains unclear, as does the true diversity of pterodactyloids from the Cretaceous of northeast Australia.

In this paper, we describe a new partial pterosaur skeleton from Australia—the most complete exemplar reported from the continent to date. It derives from the upper Lower Cretaceous Toolebuc Formation (Queensland, northeast Australia), and consists of a partial skull and complete mandible, hyoid apparatus, and partial postcranial skeleton. The new specimen is compared with other related taxa, its phylogenetic position is assessed, and its feeding habit discussed based on its cranial, dental, and hyoid morphology.

## Geological setting

**Figure 1:**
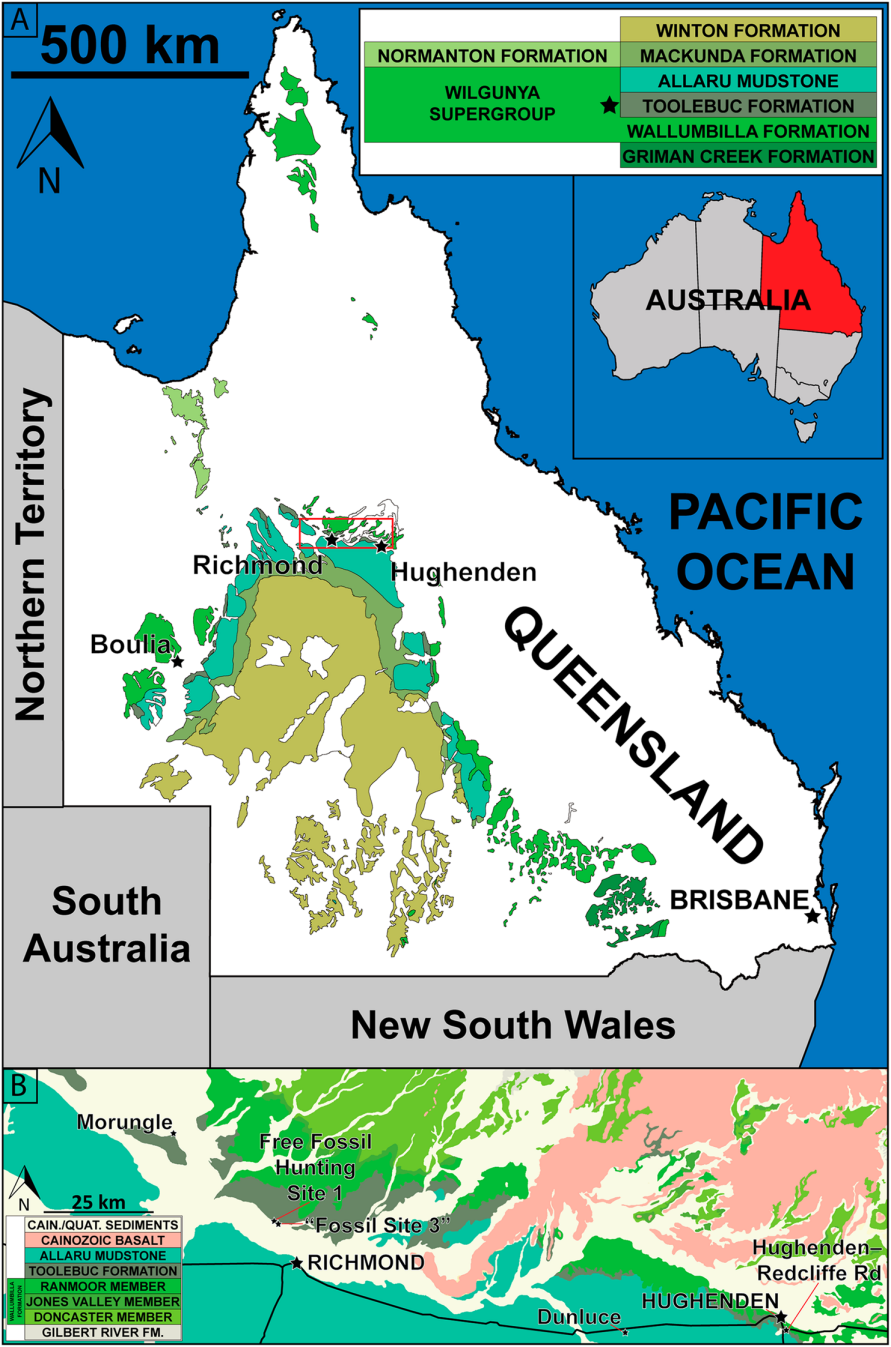
(**A**) Map of Queensland showing the extent of Cretaceous outcrop. (**B**) Map of the location of Dig Site Three, and numerous other sites in the area from which pterosaur fossils have been collected. Map drafted by S.F.P. in Adobe Illustrator CC 2024 (modified from Poropat, et al.^33,34^, incorporating geological outcrop data from Vine, et al.^35^ and Vine and Paine^36^ [ © Commonwealth of Australia (Geoscience Australia) 2024. This product is released under the Creative Commons Attribution 4.0 International Licence. http://creativecommons.org/licenses/by/4.0/legalcode].

The partial pterosaur skeleton described herein (KK F1426) was collected by K. Petersen in November 2021 at a site dubbed Dig Site 3 (Fig. 1), from exposures ascribed to the Toolebuc Formation (middle–upper uppermost Albian, corresponding to the *Pseudoceratium ludbrookiae* and *Phimopollenites pannosus* spore/pollen biostratigraphic zones^28–30^). Other remains associated with the KK F1426 specimen include bivalve molluscs (*Inoceramus sutherlandi*, *Aucellina hughendensis*) and indeterminate actinopterygian fish remains. The Toolebuc Formation is hosted within the Eromanga Basin, with the latter comprising Jurassic–Cretaceous sediments, with surface exposures across a large portion of Queensland, and extending into the Northern Territory, South Australia and New South Wales^31,32^.

**Figure 2:**
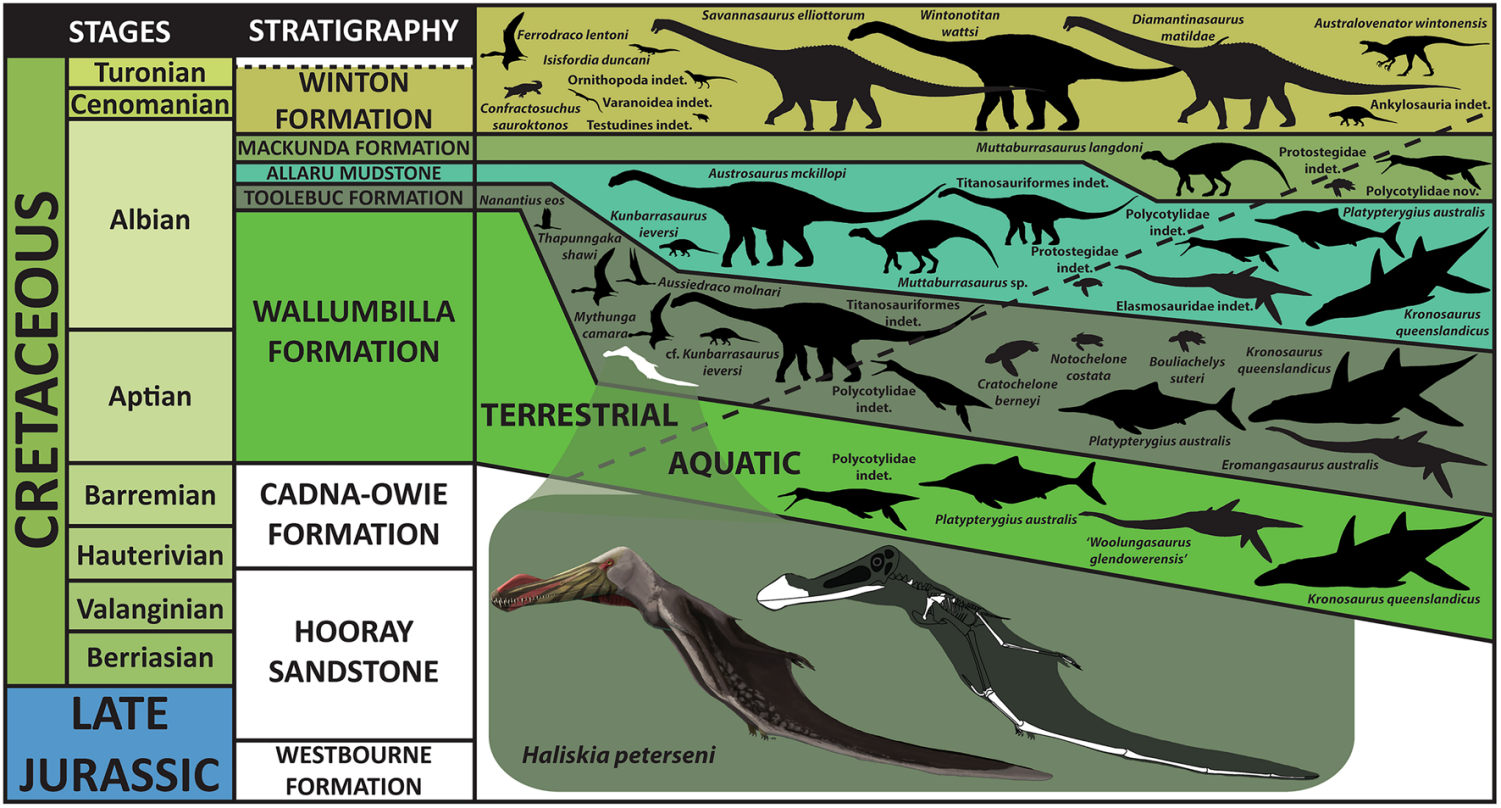
Stratigraphy of the Eromanga Basin, Queensland, Australia. Silhouettes of tetrapod taxa used herein by Stephen Poropat, Travis Tischler, Julius Csotonyi, Ken Kirkland, Steve Kirk, Dmitry Bogdanov, Ray Chatterji, Mike Keesey, and Gabriel Ugueto. Restoration of *Haliskia peterseni* by R.J.D. and skeletal reconstruction by A.H.P. based on *Tropeognathus mesembrinus* by Witton^45^. Modified from Poropat, et al.^46^.

The lithology of the Toolebuc Formation comprises mixed limestones and oil shales, with sediments deposited during flooding within an anoxic and partially restricted basin, during rapid and renewed marine transgression during the Albian^37–39^. The vertebrate assemblage of the Toolebuc Formation is diverse and abundant, comprising plesiosaurs (including pliosaurids, elasmosaurids and polycotylids), the ichthyosaur *Platypterygius australis *McCoy 1867^40^, protostegid sea turtles, several chondrichthyan and actinopterygian fishes, as well as remains from volant enantiornithine birds, pterosaurs and rare terrestrial non-avian dinosaurs (Fig. 2)^8,25^.

## Results

### Systematic palaeontology

PTEROSAURIA Kaup, 1834^41^

PTERODACTYLOIDEA Plieninger, 1901^42^

PTERANODONTOIDEA Marsh, 1876^43^ sensu Kellner, 2003^44^

ANHANGUERIA sensu Rodrigues and Kellner, 2013^45^

ANHANGUERIDAE Campos and Kellner, 1985^46^

*Haliskia peterseni* gen. et sp. nov.

*Type Species. Haliskia peterseni* gen. et sp. nov.

*Diagnosis*. As for species.

**Figure 3:**
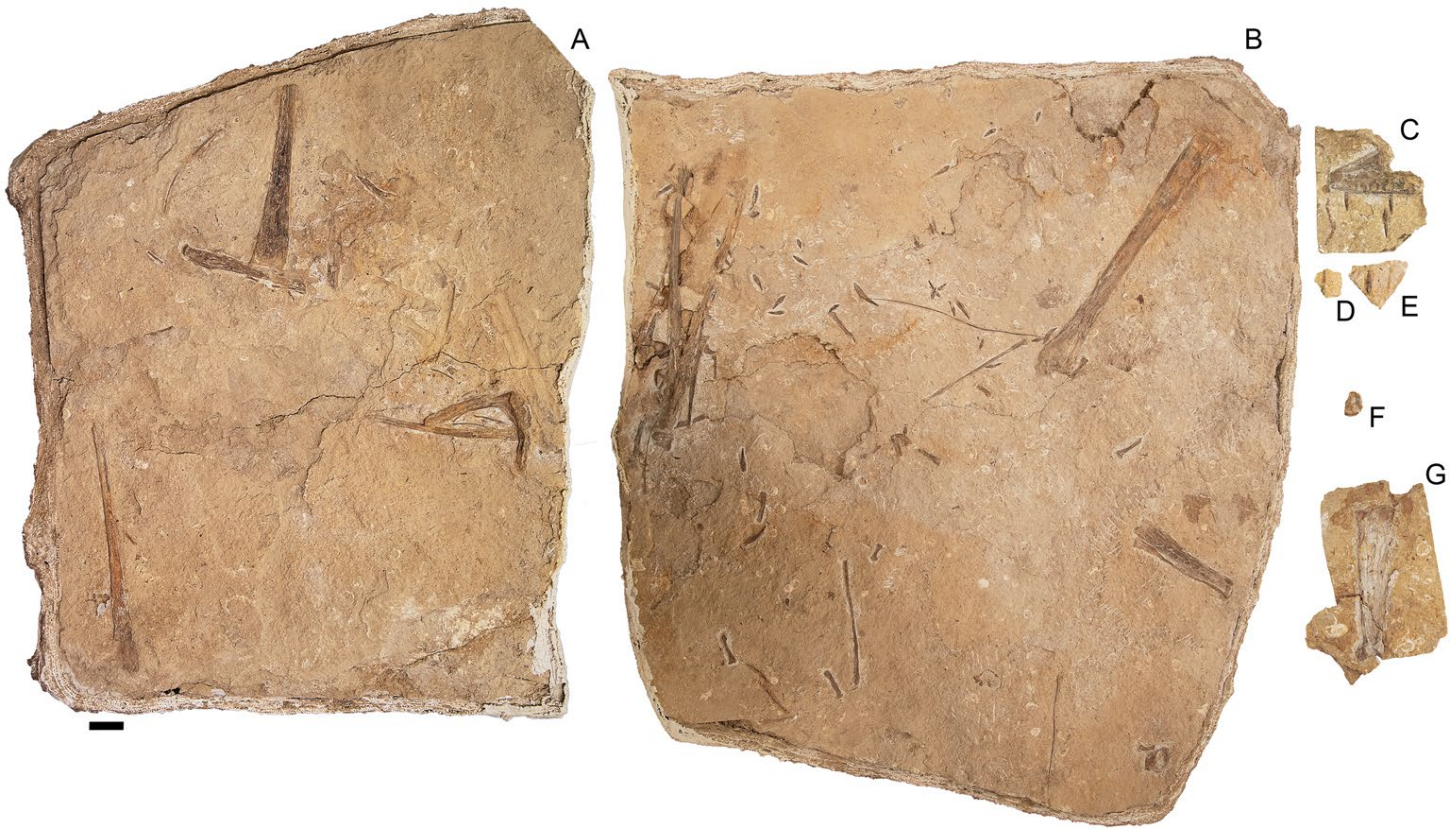
*Haliskia peterseni* gen. et sp. nov., holotype specimen KK F1426. (**A**) dorsal vertebra, ribs, gastralium, left scapulocoracoid, left and right manual phalanx IV-2, right manual phalanx IV-3, left femur, left tibia, metatarsals and pedal phalanges, (**B**) mandible, teeth, ceratobranchials, cervical vertebra, rib, gastralium, right syncarpus, right lateral carpal, right pteroid, metacarpals, manual digits, right manual phalanx IV-1, left manual phalanx IV-4, (**C**) premaxilla with teeth, (**D**) isolated tooth, (**E**) isolated tooth, (**F**) left lateral carpal, (**G**) left metacarpal IV. Scale bar = 50 mm.

**Figure 4:**
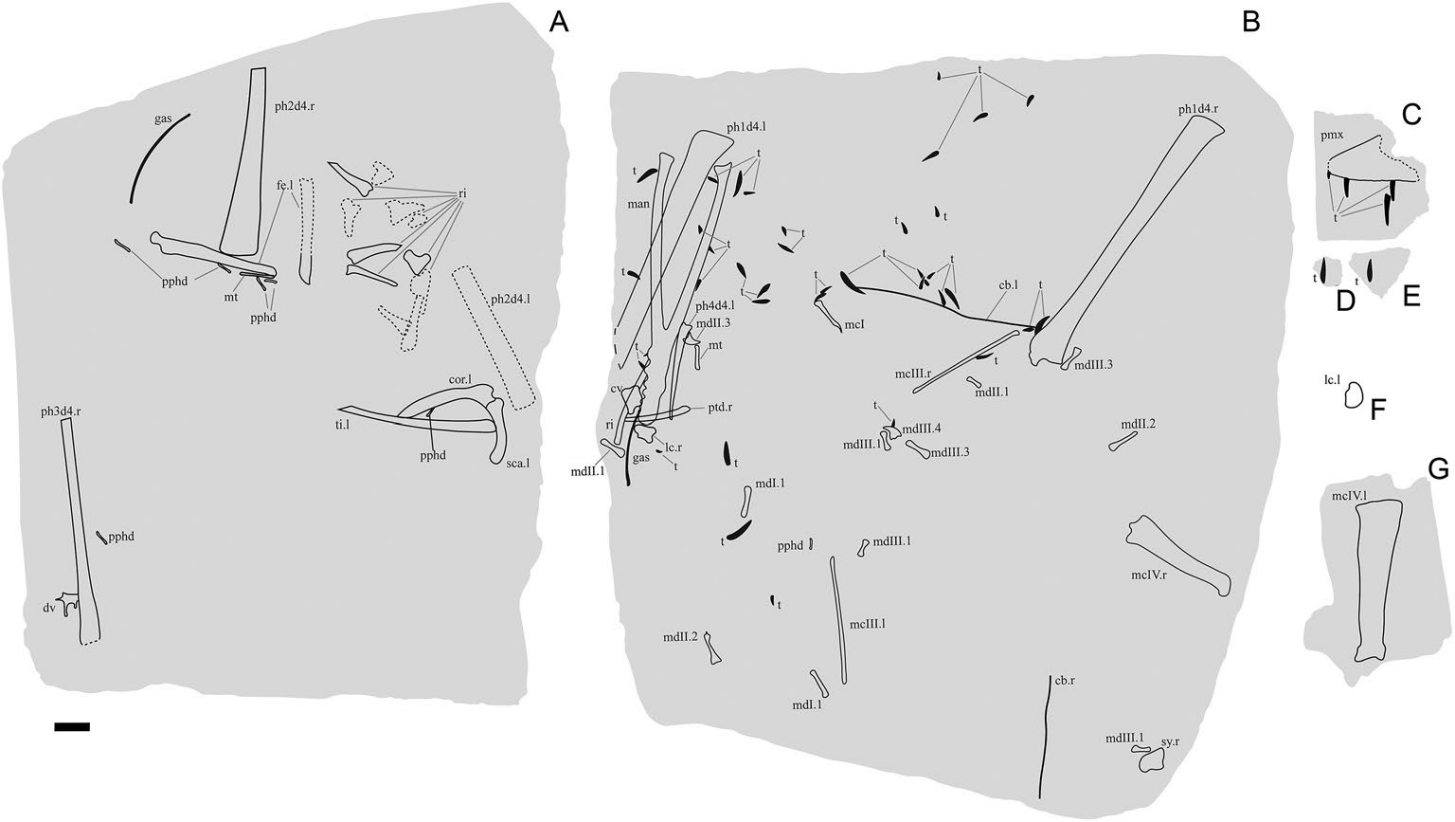
*Haliskia peterseni* gen. et sp. nov., schematic drawing of holotype specimen KK F1426. (**A**) dorsal vertebra, ribs, gastralium, left scapulocoracoid, left and right manual phalanx IV-2, right manual phalanx IV-3, left femur, left tibia, metatarsals and pedal phalanges, (**B**) mandible, teeth, ceratobranchials, cervical vertebra, rib, gastralium, right syncarpus, right lateral carpal, right pteroid, metacarpals, manual digits, right manual phalanx IV-1, left manual phalanx IV-4, (**C**) premaxilla with teeth, (**D**) isolated tooth, (**E**) isolated tooth, (**F**) left lateral carpal, (**G**) left metacarpal IV. Abbreviations: cb, ceratobranchial; cor, coracoid; cv, cervical vertebra; dv, dorsal vertebra; fe, femur; gas, gastralium; lc, lateral carpal; l, left; man, mandible; mcI–IV, metacarpals I–IV; mdI–III, manual digits I–III;  mt, metatarsal; ph1–4d4, phalanges of manual digit IV; pmx, premaxilla; pphd, phalanges of pedal digit; ptd, pteroid; r, right; ri, rib; sca, scapula; sy, syncarpus; t, teeth; ti, tibia. Scale bar = 50 mm.

*Holotype specimen.* KK F1426: anterior portion of skull including partial premaxillary crest; complete mandible; both ceratobranchials; one cervical vertebra, one dorsal vertebra; twelve dorsal ribs; two gastralia; left scapulocoracoid; right syncarpus; both lateral carpals; partial right pteroid; right metacarpals I–III; non-flight digit manual phalanges and unguals; left and right metacarpal IV; left and right manual phalanges IV-1; partial right manual phalanx IV-2; partial right manual phalanx IV-3; partial left manual phalanx IV-4; 43 teeth; left femur; left tibia; two metatarsals; seven pedal phalanges; and associated fragments (Figs. 3, 4).

*Horizon and locality*. Toolebuc Formation, middle–upper uppermost Albian^28^; Dig Site 3, NW of Richmond, Queensland, Australia.

*Etymology*. The genus epithet (pronounced ‘hay-li-sky-ah’) *Haliskia* derived from Ancient Greek *ἅλς* (háls) = ‘sea’; and *σκῐᾱ́* (skiā́) = shadow, phantom, or evil spirit; thus, a flying creature that cast a shadow on the sea, or a phantom that haunted the long-vanished Eromanga Sea.

The species epithet honours Kevin Petersen, who recovered and prepared the specimen.

*Diagnosis*. *Haliskia peterseni* can be distinguished from other anhanguerians by the following combination of characters (autapomorphies marked with an asterisk): anterior margin of the premaxilla flattened; anterior portions of jaws not laterally expanded; alveolar borders inflated relative to jawline; subtle palatal ridge which begins at the 2nd tooth pair and extends until the 8th; premaxillary crest level with anterior margin of skull, rises steeply at an angle of 30°; comparatively short mandibular crest; ceratobranchial: skull length ratio 70%*; 4th and 5th teeth smaller than 3rd and 6th*; 2nd and 5th alveoli smaller than pairs 3–4 and 6–8*; marked increase in interalveolar spacing after the 6th alveoli.

### Description

#### Premaxilla

**Figure 5:**
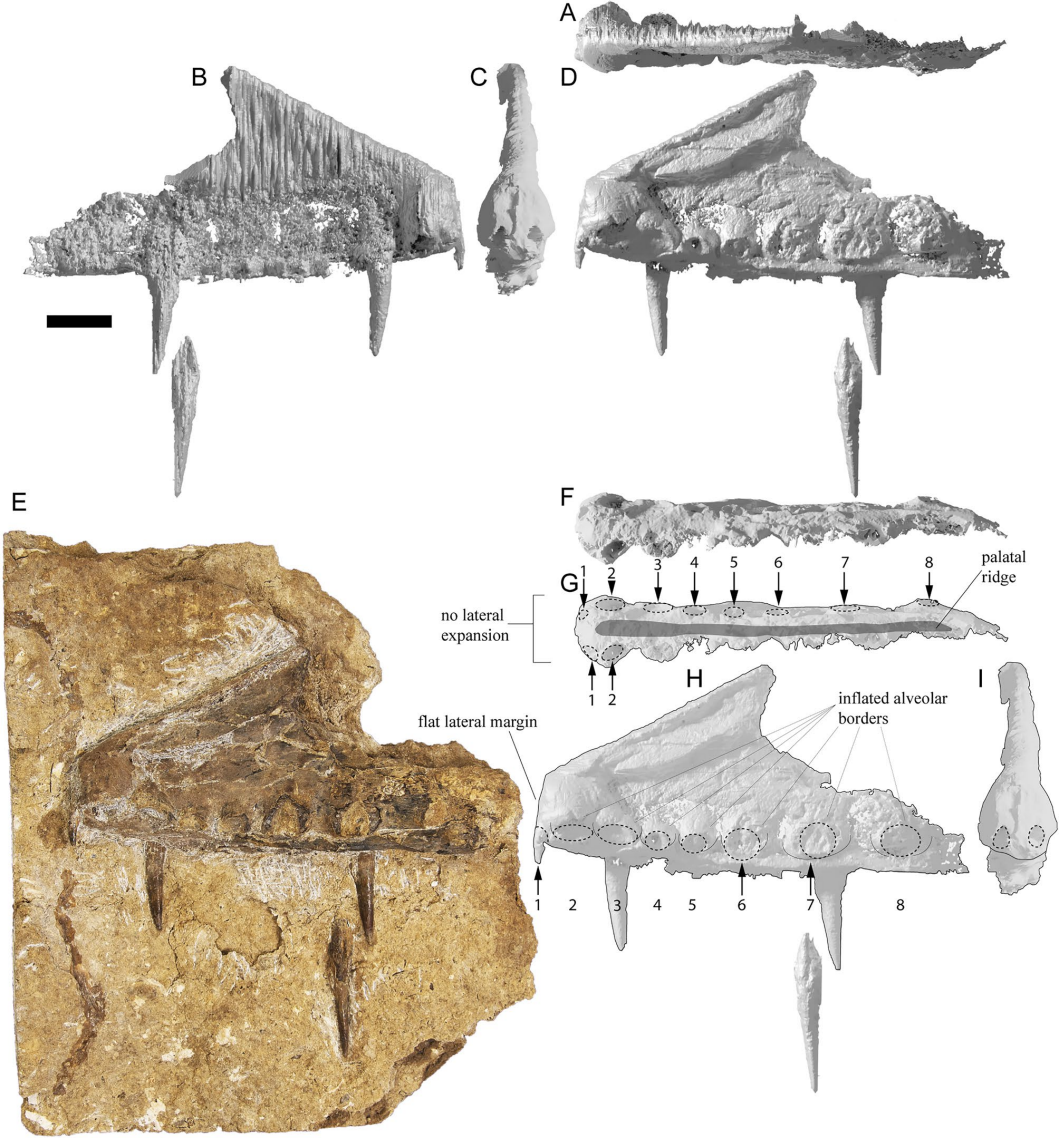
*Haliskia peterseni* gen. et sp. nov., holotype premaxilla of KK F1426 in (**A**) dorsal, (**B**) right lateral, (**C**) anterior, (**D**) and (**E**) left lateral, (**F**) and (**G**) ventral, (**H**) left lateral and (**I**) anterior view. A–D & F–I are renders of digital models generated from neutron scans, E is a photograph. Numbers 1–8 indicate alveoli, alveoli position indicated with dashed line. Scale bar = 20 mm.

The skull of KK F1426 is represented by the anterior end of the premaxilla and premaxillary crest, visible in left lateral view and preserved on a separate slab from the appendicular elements (Figs. 3C, 4C, 5). Four teeth are preserved with the premaxilla, with in situ teeth occupying the first alveolus on the left, and the 3rd and 7th alveoli on the right. The remaining tooth was dislodged from, and is located near the 7th alveolus. The preserved teeth show a smooth enamel that lacks ornamentation.

In lateral view, the tooth row is straight, except for a ventrally deflected protuberance visible dorsal to the 5th and 6th alveoli, which is the result of post-mortem crushing to the right margin. Based on thermal-neutron computed tomography (NCT), the rostrum is not anteriorly expanded and the palatal ridge initiates with the 2nd alveolus and extends beyond the 8th alveolus (Fig. 5F–G).

**Figure 6:**
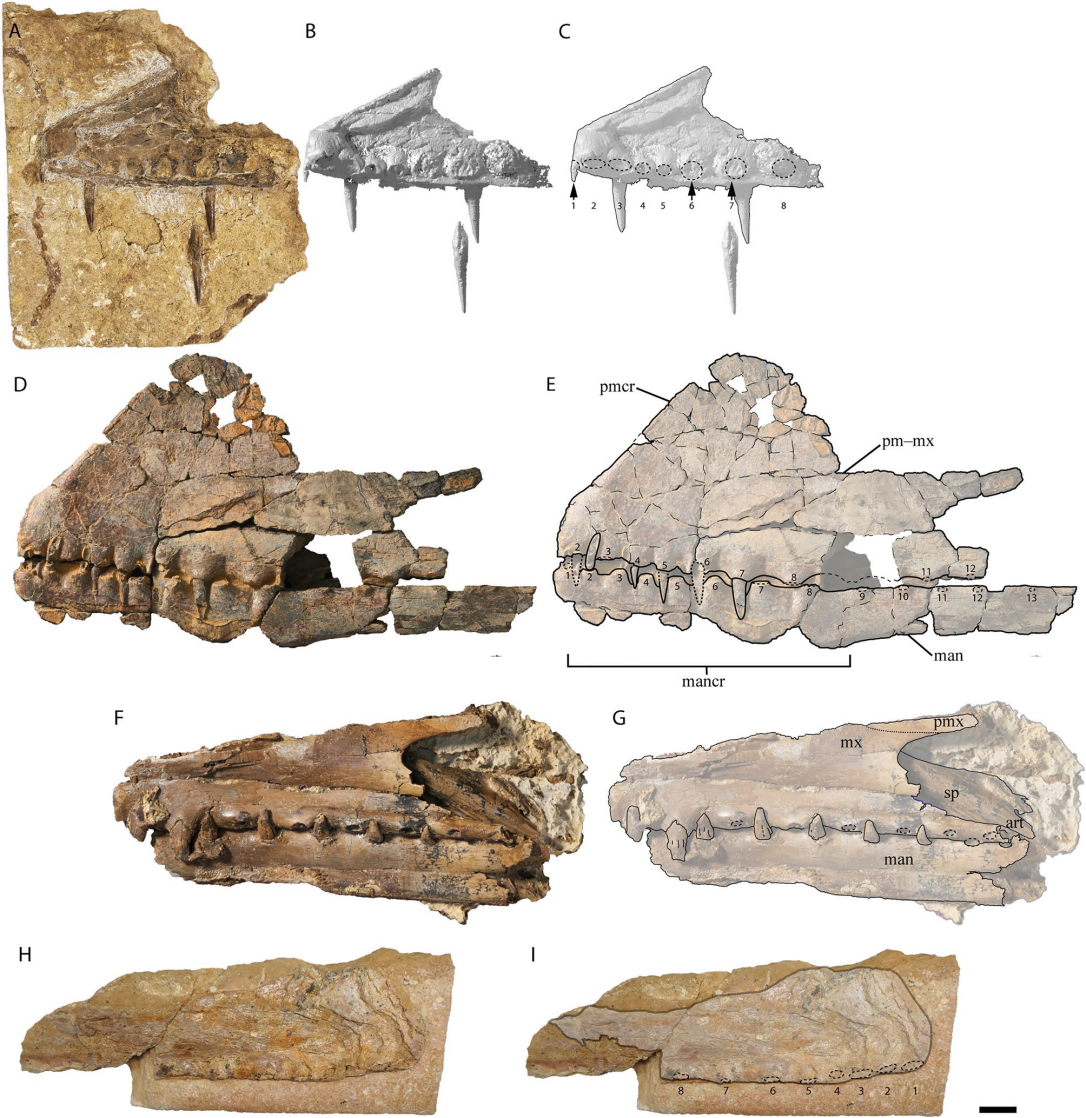
Australian pterosaur cranial material. (**A**), (**B**) and (**C**) *Haliskia peterseni *gen. et sp. nov., holotype premaxilla and teeth; (**D**) and (**E**) *Ferrodraco lentoni* holotype premaxilla–maxilla and mandible (AODF 876); (**F**) and (**G**) *Mythunga camara *holotype skull and mandible (QM F18896); (**H**) and (**I**) KK F600 premaxilla. A, D–I are photographs, B–C are renders of a digital model generated from neutron scans. A, D–E taken by A.H.P., B–C by R.J.D, F–G taken by S.F.P. and H–I taken by Michelle Johnstone. Scale bar = 20 mm.

In lateral view, the anterior margin of the premaxilla is flat and bears a confluent premaxillary crest. The anterior margin of the premaxilla bears a subtle, shallow depression similar to *Ornithocheirus* cf. *simus* (FSAC-KK 5025) and *Coloborhynchus* sp. (FSAC-KK 5024^47^); however, it is possible this feature is the result of post-mortem crushing. Although the morphology of the premaxillary crest alone cannot be used to distinguish KK F1426 from other anhanguerians^5^, the straight dorsal margin and relatively shallow incline is reminiscent of the premaxillary crests of *Siroccopteryx moroccensis*^48^ and *Ferrodraco lentoni*^11,12^. However, the angle of the premaxillary crest in KK F1426 is shallower when compared with that observed in *Ferrodraco*^12^ (Fig. 6). Moreover, the premaxillary crest is transversely wider in *Haliskia* than in *Ferrodraco*, despite the former showing clear evidence of mediolateral flattening (Fig. 5C). As preserved, the premaxillary crest is smooth, unornamented, and mediolaterally thin (4 mm in width, based on synchrotron scan data).

**Figure 7:**
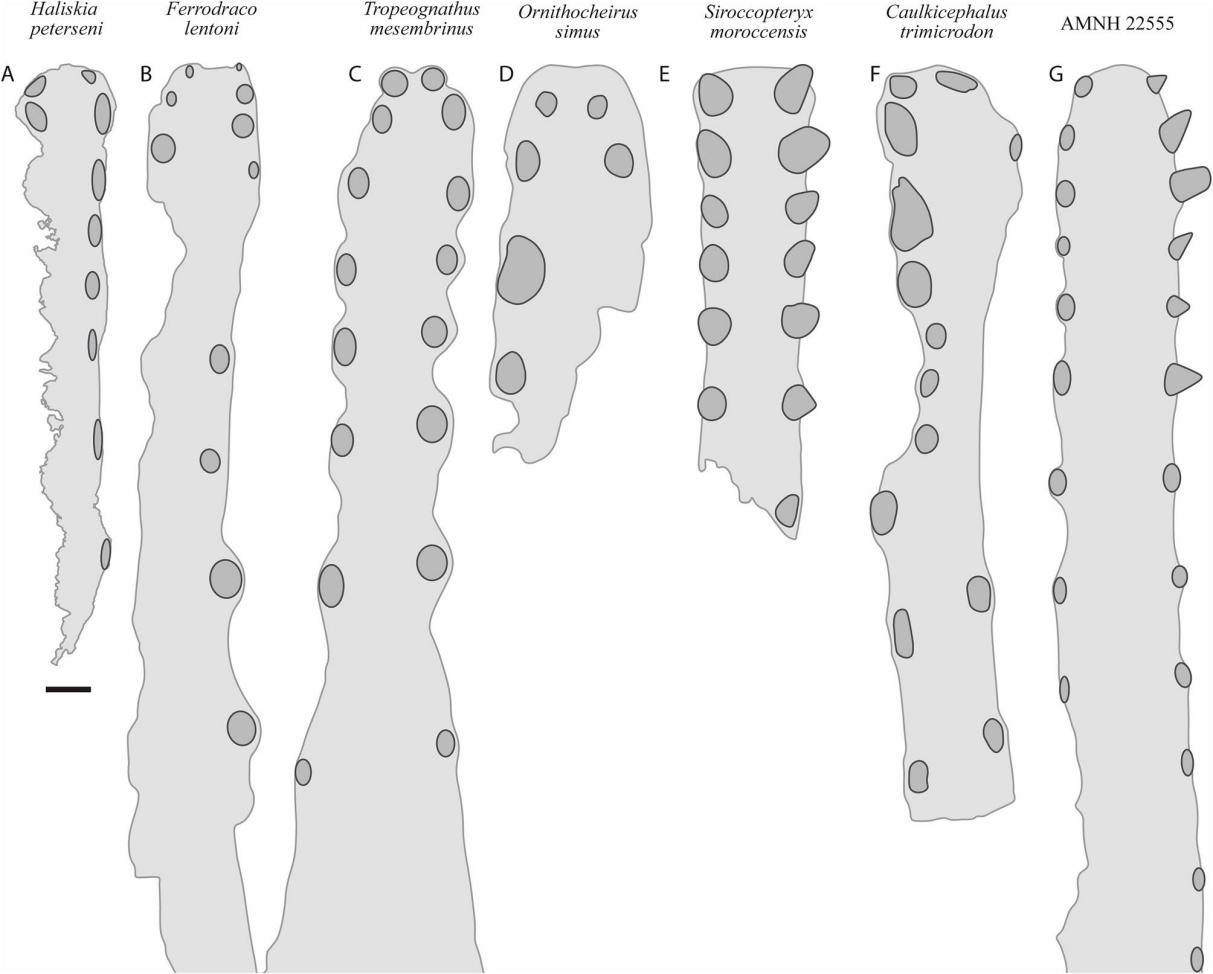
Alveolar configuration of anhanguerian pterosaurs. (**A**) *Haliskia peterseni *gen. et sp. nov., holotype in occlusal view; (**B**) *Ferrodraco lentoni *holotype premaxilla–maxilla (AODF 876) in occlusal view redrawn from Pentland, et al.^12^; (**C**) *Tropeognathus mesembrinus* (BSP 1987 I 46) in occlusal view redrawn from Pentland, et al.^12^; (**D**)* Ornithocheirus simus* (CAMSM B54428) in occlusal view redrawn from Rodrigues and Kellner^51^; (**E**) *Siroccopteryx moroccensis *holotype (LINHM 016) in occlusal view redrawn from Mader and Kellner^48^; (**F**) *Caulkicephalus trimicrodon *holotype (IWCMS 2002.189.1, 2, 4) in occlusal view redrawn from Steel, et al.^52^; (**G**) AMNH22555 in occlusal view redrawn from Wellnhofer^53^. Scale bar = 10 mm.

The preserved section of the rostrum hosts eight alveoli (Figs. 5G–H, 7) Table S1). The alveoli have distinct borders, as in *Mythunga camara*^19,25^ and *Ferrodraco lentoni*^12^ (Fig. 6). Based on CT scan data, the anteriormost alveoli are vertical, and level with the rest of the toothrow. Moreover, the first six alveoli are evenly and closely spaced, with the mesiodistal length between successive alveoli (interalveolar spacing) increasing posterior to the sixth alveolus (Table S2). The interalveolar spacing in the preserved section of the premaxilla is greater than the diameter of the alveolus. Although the premaxilla is still partially embedded in matrix, the presence of a tooth in the first alveolus indicates that the first tooth pair of the premaxilla was positioned dorsally relative to the tooth row (Fig. 5I); this feature is shared with *Tropeognathus mesembrinus*^49^ and *Ferrodraco lentoni*^12^. *Haliskia peterseni* differs from other anhanguerians, in that the 4th and 5th teeth are smaller than 3rd and 6th. By contrast, in *Anhanguera*, the 5th and 6th teeth are smaller than the 4th and 7th^5^. *Haliskia* can be distinguished from KK F600, based on the alveolar configuration, wherein the first four alveoli are spaced close together in the latter. Although KK F600 was referred to *T. shawi* by Richards, et al.^26^, we question this referral based on the lack of anatomical overlap between the holotype and referred specimen, the presence of at least three pterosaur taxa in the Toolebuc Formation and the taxonomic diversity of anhanguerians in other localities, such as the Araripe Basin^8^.

#### Mandible

**Figure 8:**
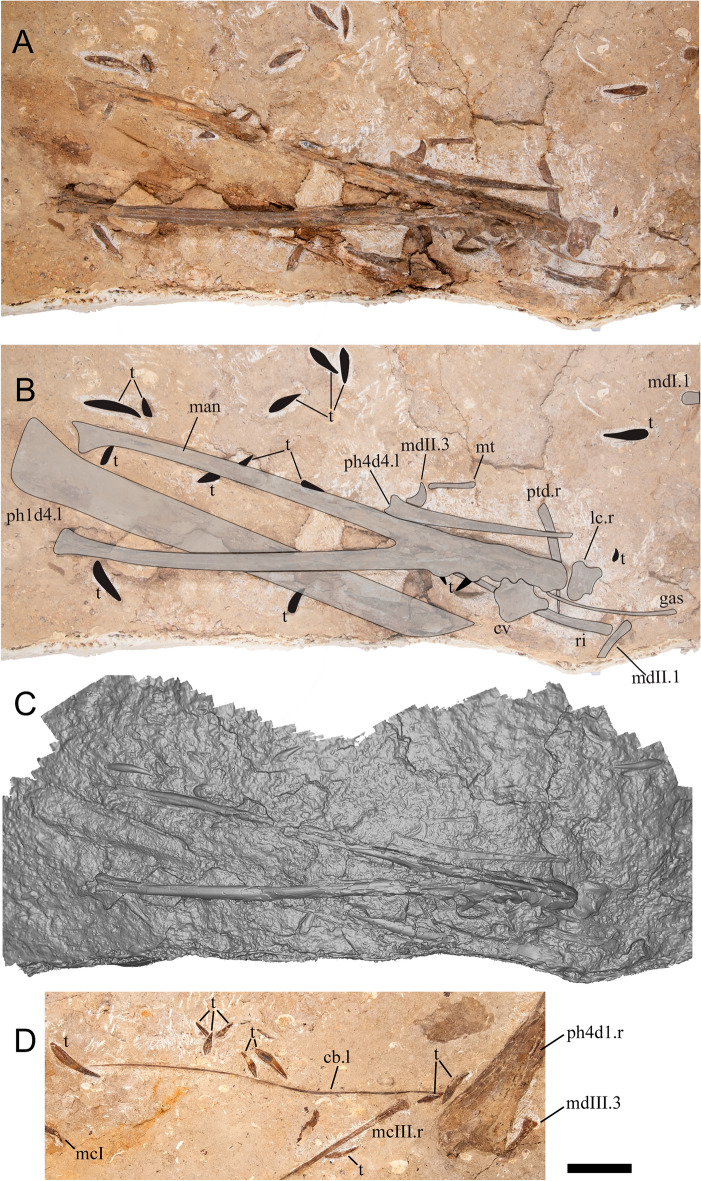
*Haliskia peterseni* gen. et sp. nov., holotype mandible and left ceratobranchial. **A**, **B** and **D** photographs, and **C** is a three-dimensional surface render. Abbreviations: cb, ceratobranchial; cv, cervical vertebra; gas, gastralium; l, left; lc, lateral carpal; man, mandible; mdI–mdIII, manual digits I–III; mt, metatarsal; ph4d1, first phalanx of manual digit IV; ph4d4, fourth phalanx of manual digit IV; ptd, pteroid; r, right; ri, rib; t, teeth. Scale bar = 50 mm.

**Figure 9:**
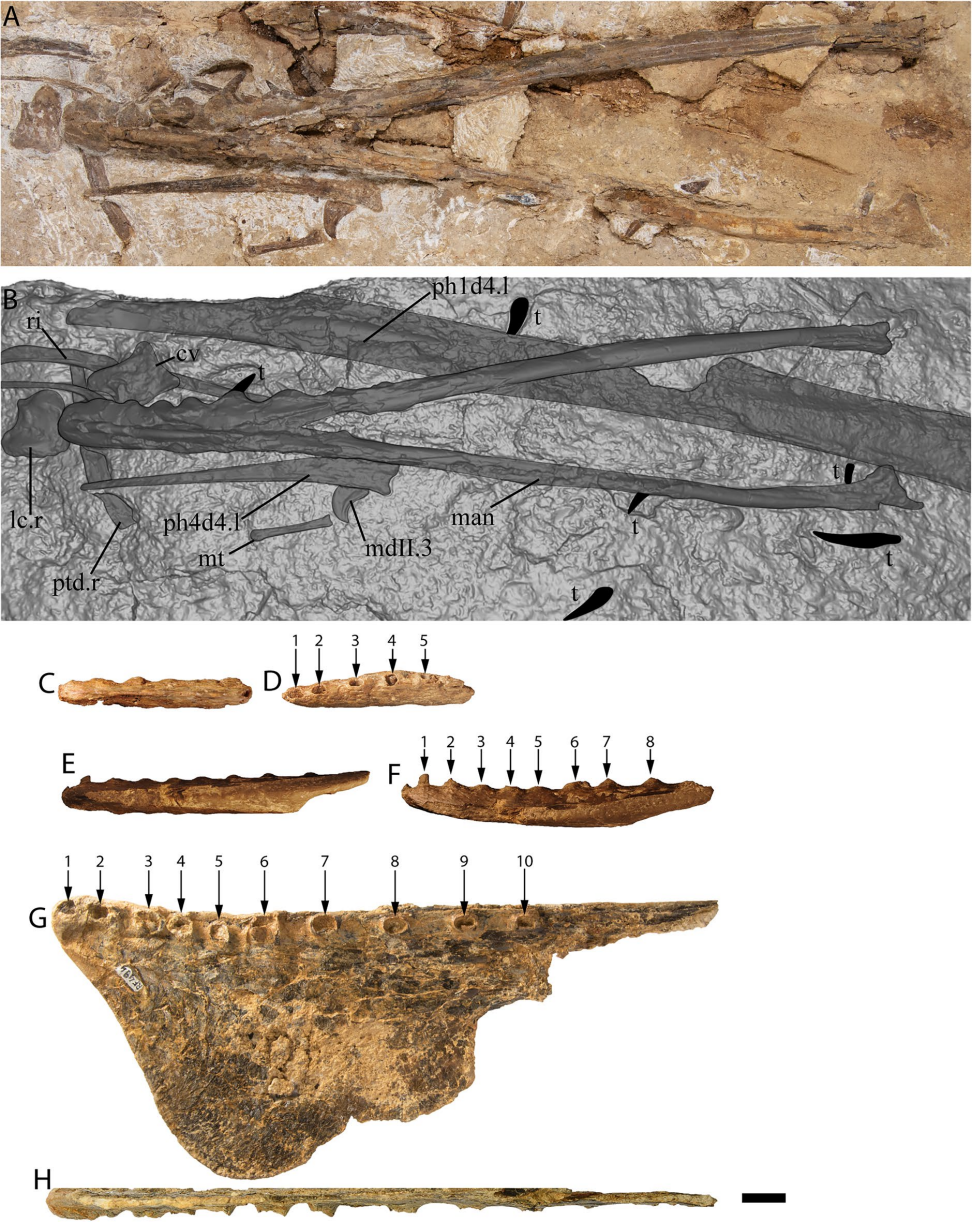
Australian pterosaur mandibles. (**A**) and (**B**) *Haliskia peterseni* gen. et sp. nov., holotype mandible (KK F1426); (**C**) and (**D**) *Aussiedraco molnari *holotype mandible (QM F10613), (**E**) and (**F**) QM 44423 mandible; (**G**) and (**H**) *Thapunngaka shawi* holotype mandible (KK F494). A, C–H, are photographs, B is a render of a digital model generated from neutron scans. A–B and G–H taken by A.H.P., C–F photographs taken by S.F.P. Scale bar = 20 mm.

The mandible is complete and consists of the fused mandibular symphysis, and both mandibular rami preserved in ventral view; only the left retroarticular process is missing (Figs. 8, 9). Based on the morphology of the occlusal surface of the premaxilla, the occlusal surface of the mandible is most likely relatively straight, thus differing from *Aussiedraco molnari*^22^. The anterior part of the mandible is not transversely expanded, and the mandibular crest is restricted to the anterior third of the lower jaw. Unfortunately, the mandibular crest suffered damage during excavation, such that it is incomplete posteriorly. Moreover, the fact that the mandibular crest is not positioned on the midline implies that it has been distorted by taphonomic processes. Nevertheless, the mandibular crest was clearly short dorsoventrally and blade-like. This distinguishes *Haliskia peterseni* from *Aussiedraco molnari* and QM F44423 which lack mandibular crests^8^, as well as the holotype specimen of *Thapunngaka shawi*^23^ which posseses a dorsoventrally tall crest (Fig. 9). As preserved, the mandibular crest is 80 mm long anteroposteriorly, 10 mm tall dorsoventrally, and extends to the level of the posterior margin of the 6th alveolus; well before the mandibular symphysis, as in *Tropeognathus mesembrinus*^49^ but contrasting with *Anhanguera spielbergi*^50^. The mandible is 400 mm long anteroposteriorly, as measured from the anterior margin to the posterior end of the right articular. The dentaries are firmly fused, forming the mandibular symphysis, which measures 133 mm anteroposteriorly (measured from the anterior tip to the posterior margin of the symphysis). The maximum width of the mandibular symphysis is 36 mm. This is transversely wider than *Aussiedraco molnari*, QM F44423 and the holotype of *Thapunngaka shawi* (KK F494); however, the latter is severely crushed^8^ (Fig. 9). The symphysis represents one-third the total length of the lower jaw, with its anteroposterior length approximately four times its lateral width. Given that the mandible is preserved in ventral view, the presence of a mandibular groove cannot be determined; however, the presence of a narrow palatal ridge on the upper jaw (based on neutron scan data) implies that KK F1426 had a mandibular groove.

Although both mandibular rami are preserved, the left is better preserved than the right; however, only the posterior end of the right ramus preserves the articular. Although it is likely that KK F1426 had a helical jaw joint (based on the triangular shape of the retroarticular process), the preservation of the mandible in ventral view precludes this from being demonstrated unequivocally. Relative to the long axis of the mandible, the retroarticular process is sub-horizontal.

In ventral view, the lateral margins of the mandibular symphysis undulate, because of the inflated alveolar borders (Fig. 8B). On the left side, the sixth alveolus is occupied by an in situ tooth, and an isolated tooth is present near the seventh alveolus. Based on the inflated alveolar borders and comparisons with the premaxilla, the mandibular symphysis bears eight alveoli on each side before the two rami diverge. Based on comparisons with *Ferrodraco*, *Mythunga*, and other anhanguerians^11,49, 54^ the degree of alveolar border inflation decreases posteriorly, and thus the total number of alveoli along the mandibular rami cannot be estimated.

#### Hyoid apparatus

Both ceratobranchials are preserved. The left ceratobranchial lies adjacent to the right manual phalanx IV-1 (Fig. 8) and the right ceratobranchial is situated between the right syncarpus and metacarpal III (Fig. 4B). The right ceratobranchial is incomplete both anteriorly and posteriorly; as such, the following description is based on the left. The ceratobranchials are unfused, despite complete fusion of the scapulocoracoid and extensor tendon process to manual phalanx IV-1. Given that ceratobranchials are rarely preserved, it remains unclear whether they fuse during the latest stage of ontogeny, or if the ceratobranchials remain unfused into adulthood in some pterosaur taxa (as suggested by Jiang, et al.^55^). The ceratobranchials of KK F1426 are less than 2 mm in diameter, relatively straight anteriorly, and gently curved laterally at the distal end. In life, the anterior ends of the ceratobranchials would have articulated such that they defined a Y-shape, as in *Ludodactylus sibbicki*^56^ and some istiodactylids^55^.

The hyoid apparatus has been observed in several anhanguerians, including *Anhanguera spielbergi*^50^, *Ludodactylus sibbicki*^56^ and *Guidraco venator*^57^. A hyoid apparatus was also noted in the more distantly related istiodactyliforms *Mimodactylus libanensis*^58^ and *Haopterus gracilis*^59^, and the lonchodectid *Ikrandraco avatar*^60^. Unfortunately, the ceratobranchials of *Anhanguera spielbergi*, *Guidraco*, *Ikrandraco* and *Haopterus* are incomplete. Although the ceratobranchials of *Ludodactylus* are complete, the lengths of these elements were not explicitly stated. Nevertheless, based on figures of the specimen, including their curvature, the ceratobranchials of *Ludodactylus* are approximately 250 mm anteroposteriorly^56^ whereas the mandible is approximately 447 mm. These estimates are concordant with the ceratobranchial: skull length ratio of 56.6% reported for *Ludodactylus* by Jiang, et al.^55^. The complete left ceratobranchial of KK F1426 is slightly longer (280 mm anteroposteriorly) than that of *Ludodactylus*, whereas the mandible is shorter (400 mm; Table 1). This results in a ceratobranchial: skull length ratio of 0.70, which is significantly longer than that of any pterosaur hitherto reported^55^. The second greatest ceratobranchial: skull length ratio (0.638) is that of the non-pterodactyloid *Dorygnathus banthensis* (SMNS 50702)^55^.

**Table 1:** Measurements of *Haliskia peterseni* (KK F1426) in millimetres.

Element	Anteroposterior Length	Transverse width	Dorsoventral height			
Upper jaw	130	15*	65*			
Lower jaw	400	36	*			
Mandibular symphysis	133	-	*			
Cervical vertebra	37	31*	-			
Dorsal vertebra		31*				

Element	Proximodistal Length	Maximum width	Dorsoventral height	Length	Maximum width	Dorsoventral height
Side	Left			Right		
Ceratobranchials	280	2		200	2	
Dorsal Rib A				139*	16	
Dorsal Rib B				84*	20	
Dorsal Rib C				111*	10*	
Dorsal Rib D				50*	22*	
Dorsal Rib E				34*	30	
Dorsal Rib F				50*‡	32	
Dorsal Rib G				4*‡	10*‡	
Dorsal Rib H				25*‡	35	
Dorsal Rib I				16*	24*	
Dorsal Rib J				68*‡	32*‡	
Dorsal Rib K				50*‡	25*‡	
Dorsal Rib L				64*‡	19*‡	
Gastralium A				113	4	
Gastralium B				138	4	
Scapula	88*					
Coracoid	145*					
Syncarpus				33	51	
Lateral carpal	35	24		34	26	
Metacarpal I				52	11	
Pteroid				90	8	
Metacarpal III	195	9		193	10	
Manual phalanx I-1	48	9		48	10	
Manual phalanx I-2 (ungual)				31		16
Manual phalanx II-1	35	14		37	9	
Manual phalanx II-2	52	9		48	9	
Manual phalanx II-3 (ungual)				17*		13*
Manual phalanx III-1	32	8		28	11	
Manual phalanx III-3	41	12		40	11	
Metacarpal IV	210	51		210	51	
Manual phalanx IV-1	423*	48*		480	61	
Manual phalanx IV-2				247*	54*	
Manual phalanx IV-3				343*	33*	
Manual phalanx IV-4	144*	14*				
Femur	232†	23				
Tibia	213*	19*				
Metatarsal A	46	3				
Metatarsal B	35	7				
Pedal digit A	26	4				
Pedal digit B	18	3				
Pedal digit C	28	3				
Pedal digit D	12*	2				
Pedal digit E	14*	3				
Pedal digit F	19	4				
Pedal digit G	18	3				

Measurements based on incomplete or obscured elements indicated with an asterisk (*); reconstructed lengths indicated with a dagger (†); measurements based on impressions are marked with a double dagger (‡).

#### Dentition

Aside from the four teeth preserved with the premaxilla, an additional thirty-seven teeth were identified with the lower jaw and appendicular elements (Figs. 2B, 4B). Two additional isolated teeth were also recovered (Figs. 3D–E, 4D–E). Each tooth is conical, vertically oriented, and gently lingually recurved. Although some teeth are more lingually recurved than others, the displacement of each tooth is less than its diameter. The teeth vary in size, particularly the apicobasal height (largest = 51 mm, smallest = 11 mm, with both including the crown and root). Based on the preserved teeth, the longest tooth is 4.6 times longer than the shortest. By contrast, the largest tooth of *Ferrodraco* is three times longer than the shortest^12^, whereas in *Zhenyuanopterus* longest tooth is more than ten times the length of the shortest^61^. The basal cross-section of each tooth in KK F1426 is essentially oval (longer mesiodistally than wide labiolingually), with the apicobasal height at least four times the basal width. Although the apices of most of the teeth are preserved, few exhibit signs of tooth wear (Fig. S1).

Comparisons between the teeth of KK F1426 and other Australian pterosaur taxa are limited, since the dentitions of *Thapunngaka shawi*^23^ and *Aussiedraco molnari*^22^ are effectively unknown. Although there is limited anatomical overlap between KK F1426 and *Mythunga camara*, the teeth of *Mythunga* are comparatively robust despite being more posteriorly positioned, and also bear striations^19,25^ (Fig. 6F–G). Moreover, the teeth of *Mythunga* are more lingually recurved, with the degree of curvature increasing posteriorly^25^. The dentition of KK F1426 is most similar to that of *Ferrodraco* in that both comprise teeth that are relatively smooth with little ornamentation. However, the teeth of KK F1426 differ from those of *Ferrodraco* by being comparatively tall and straight^12^ (Fig. 6).

#### Cervical vertebra

A single cervical vertebra is preserved near the anterior end of the mandible (Figs. 3B, 4B, 8B, 9B). The vertebra is preserved in ventral view, with one exapophysis visible; the other is overlain by the mandible. Based on comparisons with *Anhanguera* sp. AMNH 22555^53^, coupled with the angle and extent of the exapophyses, we regard this cervical vertebra as an anterior postaxial cervical vertebra (III/IV/V).

#### Dorsal vertebra

A single dorsal vertebra, comprising part of the neural arch and centrum, is preserved in either anterior or posterior view, near right manual phalanx IV-3 (Fig. 3A, 4A). As preserved, the neural spine is 10 mm tall dorsoventrally and the maximum preserved width of the centrum body is 31 mm mediolaterally.

#### Dorsal ribs

**Figure 10:**
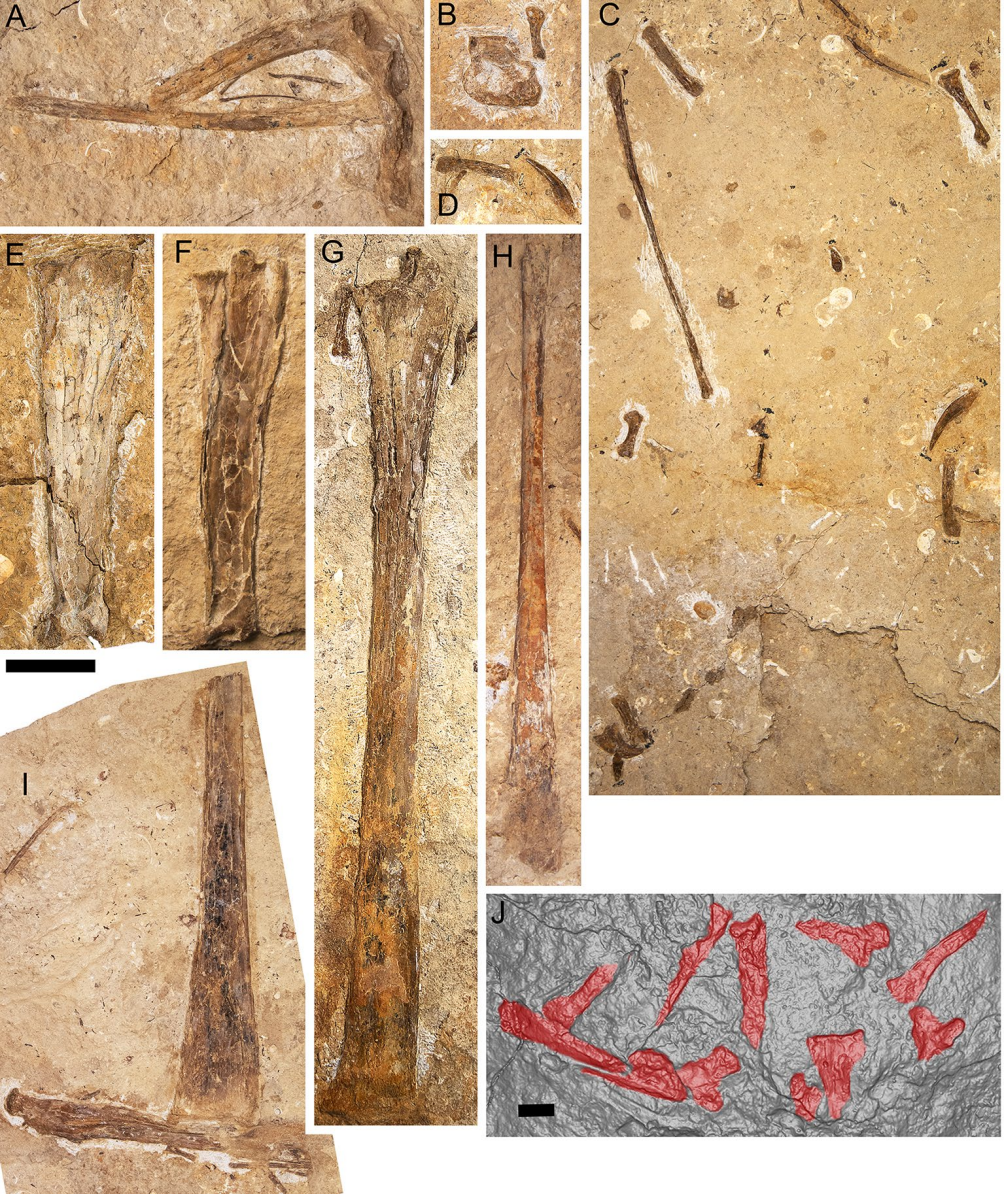
*Haliskia peterseni* gen. et sp. nov., holotype postcranial remains. (**A**) left scapulocoracoid and tibia, (**B**) right syncarpus and manual digit III-1, (**C**) right metacarpal III, isolated teeth and manual digits, (**D**) manual digit and isolated tooth, (**E**) left metacarpal IV, (**F**) right metacarpal IV, (**G**) right manual phalanx IV-1, (**H**) right manual phalanx IV-III, (**I**) right manual phalanx IV-II and left femur, (**J**) imprints from dorsal ribs. Scale bars, 50 mm in A–E, 40 mm in J.

Twelve dorsal ribs are preserved with KK F1426; however, many are represented only by impressions in the matrix (Figs. 3A, 4A, 10J, S2). Given that the precise position of the dorsal ribs cannot be stated with certainty, the most complete dorsal rib is referred to as dorsal rib A, the second most complete as dorsal rib B and so on. These ribs are dorsally arched and gently posteriorly recurved, as in *Anhanguera piscator*^54^. In dorsal rib A, the capitulum is only slightly longer than the tuberculum, and no pneumatic foramina were observed. By contrast, the tuberculum and capitulum of dorsal rib C are equidimensional.

#### Gastralia

Two gastralia are preserved: one near the left femur and right manual phalanx IV-2, the other partially overlain by the anterior end of the mandible (Figs. 3A–B, 4A–B). Given that the gastralia are anatomically identical and the precise position of these elements cannot be stated with certainty, they are referred to as gastralium A and B (Table 1). The gastralia are long, thin and dorsally recurved.

#### Scapulocoracoid

The pectoral girdle is represented by the left scapulocoracoid, which is exposed in posterior view (Figs. 3A, 4A, 10A). The scapula and coracoid are firmly fused (Fig. 10A). The distal end of each element is incomplete, such that the scapular: coracoid ratio is indeterminable. Nevertheless, it is clear that the scapula would have been shorter proximodistally (88 mm) than the coracoid (145 mm, including curvature) in vivo. The scapula is stout and constricted, with the articular surface expanded and presumably suboval, as in *Anhanguera piscator*^54^ and ‘*Anhanguera santanae*’ (AMNH 22555)^62^. By contrast, the coracoid is narrow, with the ventral margin flat. The scapula and coracoid are connected by a ‘bridge’, medial to the glenoid fossa, a feature noted in another pterosaur specimen from the Toolebuc Formation QM F10612^12,17^. As stated by Molnar and Thulborn^17^, this ‘bridge’ was also observed in *Pteranodon*^63^ and *Anhanguera*^64,53^. It cannot be determined with certainty whether the scapulocoracoid possesses a tubercle on the medial surface, distal to the coracoid process, as in *Anhanguera piscator*^54^, ‘*Anhanguera santanae*’ (AMNH 22555^62^) and QM F10612^17^. No pneumatic foramina are visible.

#### Syncarpus

The carpal series is represented by three elements. The right proximal and distal carpals are preserved together in dorsal view and lie adjacent to a manual digit (Figs. 3B, 4B, 10B). The left lateral carpal is isolated (Figs. 3F, 4F), and the right lateral carpal is anterior to the mandible and is preserved in proximal view. The morphology of the syncarpus is similar to that of *Ferrodraco lentoni*^12^ although a partial distal carpal, a sesamoid, and the lateral carpal are in situ in the latter. The syncarpus has a maximum width of 51 mm and a proximodistal length of 33 mm. The rectangular ridge on the dorsal surface is approximately 7 mm wide and 19 mm in length; however, it is incomplete. By contrast, in *Ferrodraco* the ridge on the dorsal margin is 9 mm wide and 23 mm long^12^. Given that the syncarpus is still partially embedded in matrix, the morphology of the proximal and distal articular surfaces cannot be stated with certainty. No pneumatic foramina were observed on the exposed dorsal margin of the syncarpus.

#### Pteroid

The right pteroid is preserved in medial view and comprises the diaphysis. It is 90 mm long proximodistally, and partially overlain by a dorsal rib, the anterior end of the mandible, and the left manual phalanx IV-1 (Figs. 3B, 4B, 8B, 9B). As in other pterosaurs it is gently proximally recurved, rod-like and elongated.

#### Metacarpals I–III

Right metacarpals I–III and left metacarpal III are completely preserved in KK F1426 (Figs. 3B, 4B, 10C). As in the pterodactyloid ‘*Santanadactylus pricei*’ (AMNH 22552)^53^, metacarpals I and II are reduced, whereas metacarpal III is long, thin, relatively straight, and extends along the length of metacarpal IV. The morphology of the distal articular surface of metacarpal III in KK F1426 possesses a slight ginglymoid articulation, similar to that seen in *Arthurdactylus conandoylei*^65^.

#### Manual digits I–III

Elements pertaining to the first three manual phalanges on both the left and right side are preserved, apart from four manual unguals (Figs. 3B, 4B, 10C–D). As in other pterosaurs, the phalangeal formula of digits I–III is 2-3-4, with the distal end of each penultimate phalanx terminating in a simple and slight ginglymoid articulation. The digits of KK F1426 are similar to those of *Barbosania gracilirostris* (MHNS/00/85)^66^ such that the proximal articular surfaces are approximately twice as broad dorsoventrally as the shaft.

Two manual unguals are preserved. Each is sickle-shaped and strongly recurved, with a weakly developed extensor tubercle and a prominent flexor tubercle. Based on the indeterminate pterodactyloid ‘*Santanadactylus pricei*’^53^, the flexor tubercle is most prominent in manual phalanx I-2 (the first ungual), and weakest in manual phalanx III-4 (the third ungual). The manual ungual associated with the manual phalanges is tentatively identified here as manual phalanx I-2, as the flexor tubercle is strongly developed. The other manual ungual is provisionally regarded here as manual phalanx II-3 (the second ungual), based on its proximity to the second metacarpal and digits of that element. The ratio of the depth: length of manual phalanx I-2 is 0.52. By contrast, the depth: length ratio of each ungual of *Anhanguera piscator* is ~ 0.60^54^. A weak vascular groove is present in both unguals of KK F1426. The manual unguals of KK F1426 (Table 1) are similar in size to those of *Anhanguera spielbergi*^50^^; table 20^.

#### Metacarpal IV

Both the left and right metacarpal IV are present (Table 1); however, the left metacarpal IV is preserved on a slab separate from the rest of the appendicular elements (Figs. 3G, 4G). Both elements are complete but anteroposteriorly flattened, with the left better preserved than the right. The left metacarpal IV is exposed in posterior view (Figs. 9E), whereas the right metacarpal IV is preserved in anterior view (Figs. 3B, 4B, 10E–F). Both the left and right metacarpal IV are 210 mm long proximodistally (Table 1). By contrast, the flattened but complete left metacarpal IV of *Ferrodraco* is 205 mm long proximodistally^12^, and NMV P197962, an isolated specimen described by Kellner, et al.^20^ is 212 mm long proximodistally. No pneumatic foramina were observed on either metacarpal, although this could be a consequence of crushing rather than genuine absence. As in other anteroposteriorly compressed fourth metacarpals, the distal condyles and tuberculum are less compressed when compared with the diaphysis^12,20^. As preserved, the width of distal articular surfaces are 36 mm (left) and 35 mm (right). Despite being anteroposteriorly flattened, the tuberculum of the right metacarpal IV is more pronounced with respect to the anteroventral and anterodorsal ridges, as was observed in an isolated partial metacarpal IV QM F44321;^12^. Right metacarpal IV in KK F1426 compares favorably with other pterodactyloids^54,53^, such that the tuberculum is more prominent when compared with the proximal articular face.

#### Manual phalanx IV-1

The right manual phalanx IV-1 (first wing phalanx) is visible in dorsal view (Figs. 3B, 4B, 10G). It is complete and relatively straight (Table 1), but anteroposteriorly flattened. Left manual phalanx IV-1 is incompletely preserved; much of this element is partially overlain by the mandible (Figs. 3B, 4B, 8B, 9B). The distal end of the left first wing phalanx is preserved separately, and mediolaterally compressed. As such, the follow description is based on right manual phalanx IV-1. Although the extensor tendon process is firmly fused (indicating osteological maturity), the suture between the two elements has not been completely obliterated. Pneumatic foramina were not observed along the extensor tendon process, contrasting with those observed in *Anhanguera piscator*^54^. However, this is probably because of taphonomic processes, rather than genuine absence. In dorsal view, manual phalanx IV-1 bears a large pneumatic foramen and muscle scars distal to the extensor tendon process. The position and size of these features is near-identical to that seen in *Anhanguera piscator*^54^. As preserved, the maximum width of the proximal end of the first wing phalanx is 61 mm anteroposteriorly, whereas the distal end is 54 mm. The proximal articular surface of *Haliskia peterseni* is therefore similar in size to *Anhanguera piscator* (65 mm)^54^ and ‘*Araripedactylus dehmi*’ (BSP 1975 I 166)^67^, and is longer anteroposteriorly than *Ferrodraco* (54 mm)^12^. Unfortunately, the posterior portion of the proximal articular surface is incomplete; thus, the extent of the posterior deflection cannot be determined with certainty.

#### Manual phalanx IV-2

The right manual phalanx IV-2 (second wing phalanx) is represented by the proximal articular surface and part of the diaphysis (Figs. 3A, 4A, 10I). This element is preserved in contact with the left femur. As preserved, manual phalanx IV-2 is 250 mm long proximodistally, whereas the proximal articular surface has a maximum width of 53 mm.

#### Manual phalanx IV-3

The right manual phalanx IV-3 is incomplete and represented by the diaphysis (Figs. 3A, 4A, 10H). Based on impressions in the accompanying matrix, the distal end was preserved, but lost during excavation. The morphology of the proximal articular surface cannot be characterised with certainty. As preserved, manual phalanx IV-3 is 343 mm long proximodistally with a maximum mediolateral width of 33 mm. The impression of the distal articular surface implies that it was posteriorly deflected, as in the indeterminate pterodactyloid *Santanadactylus pricei *(AMNH 22552)^53^.

#### Manual phalanx IV-4

The left manual phalanx IV-4 is represented by the diaphysis and proximal end, with its long axis parallel to the mandible (Figs. 3B, 4B, 8B, 9B). Although the proximal articular surface is partially obscured, the overall morphology compares favorably with ‘*Santanadactylus pricei*’ (AMNH 22552^53^). Manual phalanx IV-4 becomes anteroposteriorly narrower towards the distal end, as in *Anhanguera piscator*^54^.

#### Femur

The left femur is preserved in two pieces (Figs. 3A, 4A), the larger of which comprises the proximal end and diaphysis (178 mm long proximodistally), exposed in anterior view (Fig. 10I). Based on the proximodistal lengths of both pieces, we estimate that the femur was 232 mm long, similar to that of *Anhanguera piscator*^54^. The femoral head is constricted, as in other pterosaurs^50,54^, and medially deflected by 20° relative to the diaphysis, which is relatively straight. The deflection of the femoral head differs from that of *Anhanguera piscator*^54^ but is similar to an isolated specimen from the Winton Formation (AODF 2297)^24^. A proximodistally short, deep groove on the femoral head is regarded here as the fovea capitis of ligamentum teres. Although the femur has been anteroposteriorly flattened, the head is clearly hemispherical, as in *Anhanguera spielbergi*^50^ and *Anhanguera piscator*^54^. As preserved, the femur is 178 mm proximodistally, with a maximum width of 23 mm, as measured from the greater trochanter. Little can be said regarding the morphology of the greater trochanter, beyond the fact that it is ‘saddle-shaped’.

#### Tibia

The left tibia is visible in lateral view near the left scapulocoracoid and is represented by the proximal articular surface and part of the diaphysis (Figs. 3A, 4A, 10A). Including the curvature, the tibia is 143 mm proximodistally with a maximum preserved width of 19 mm. Unlike *Anhanguera piscator*, the present specimen seems to lack a tuberosity on the posteromedial surface^54^.

#### Metatarsals

Two metatarsals are preserved, one near the distal end of the left femur, the other near manual phalanx II-3 (Figs. 3A, 4A). Given that the precise position of these elements within the pes cannot be stated with certainty, they are referred to as metatarsals A and B (Table 1). Both metatarsals are anteroposteriorly elongate and uniform in thickness, except for the proximal and distal articular surfaces of metatarsal B, which are slightly expanded.

#### Pedal phalanges

Seven pedal phalanges are preserved, three pedal phalanges near the left femur (Figs. 3A, 4A, 10I), one adjacent to the femoral head, one associated with the right manual phalanx IV-3, one partially obscured by the scapulocoracoid and one near the left metacarpal III (Figs. 3, 4). Each phalanx is straight and narrow. Phalanges that preserve their articular surfaces terminate in a slight ginglymoid articular facet.

#### Phylogenetic analysis

To assess the phylogenetic placement of *Haliskia peterseni*, the new taxon was scored and included in two phylogenetic datasets. The Holgado and Pêgas^16^ dataset was chosen because it is the most up-to-date and comprehensive in terms of anhanguerian pterosaurs, whereas the Andres^68^ dataset was selected because it is the most comprehensive in terms of character scores and number of taxa included. KK F1426 can be scored for 30.7% of the characters in the Holgado and Pêgas^16^ data matrix, and 27.6% of the characters in the Andres^68^ matrix.

The initial phylogenetic analysis, based on a modified version of the dataset of Holgado and Pêgas^16^, produced 6 MPTs with a minimum length of 419 steps, minimum consistency index (CI) of 0.625, and retention index (RI) of 0.866; in this analysis, Tropeognathinae was resolved as a polytomy. *Thapunngaka shawi*^23^ was identified as an unstable operational taxonomic unit and excluded a priori; the subsequent analysis resulted in fifteen MPTs, with 417 steps, CI of 0.628 and RI of 0.867. The topology of the strict consensus tree is almost identical to that of the original tree presented by Holgado and Pêgas^16^, with *Haliskia peterseni* resolved as sister to *Mythunga* + *Ferrodraco*. This clade was resolved as sister to *Tropeognathus mesembrinus* + *Siroccopteryx moroccensis*, with the five taxa grouped together in Tropeognathinae sensu Holgado and Pêgas^16^.

The initial phylogenetic analysis by parsimony based on the Andres^68^ dataset resulted in 22 most parsimonious trees (MPTs), with a minimum length of 1373.215 steps, CI of 0.287 and RI of 0.788. The Ornithocheirae was poorly resolved and reduced to a polytomy, with *Anhanguera blittersdorffi* and *Anhanguera piscator* resolved as sister taxa. *Mythunga camara* and *Thapunngaka shawi* were subsequently identified as unstable and excluded a posteriori, resulting in a single MPT, with a tree length of 1372.215 steps, CI of 0.287 and RI of 0.788; near-identical to the results originally reported by Andres^68^. The topology of the strict consensus tree is similar to that of Andres^68^; with *Haliskia* + *Ferrodraco* resolved as sister taxa outside Ornithocheirinae, and *Tropeognathus mesembrinus* the successive sister taxon to that clade within Ornithocheiridae.

## Discussion

### Phylogenetic affinities of *Haliskia peterseni*

**Figure 11:**
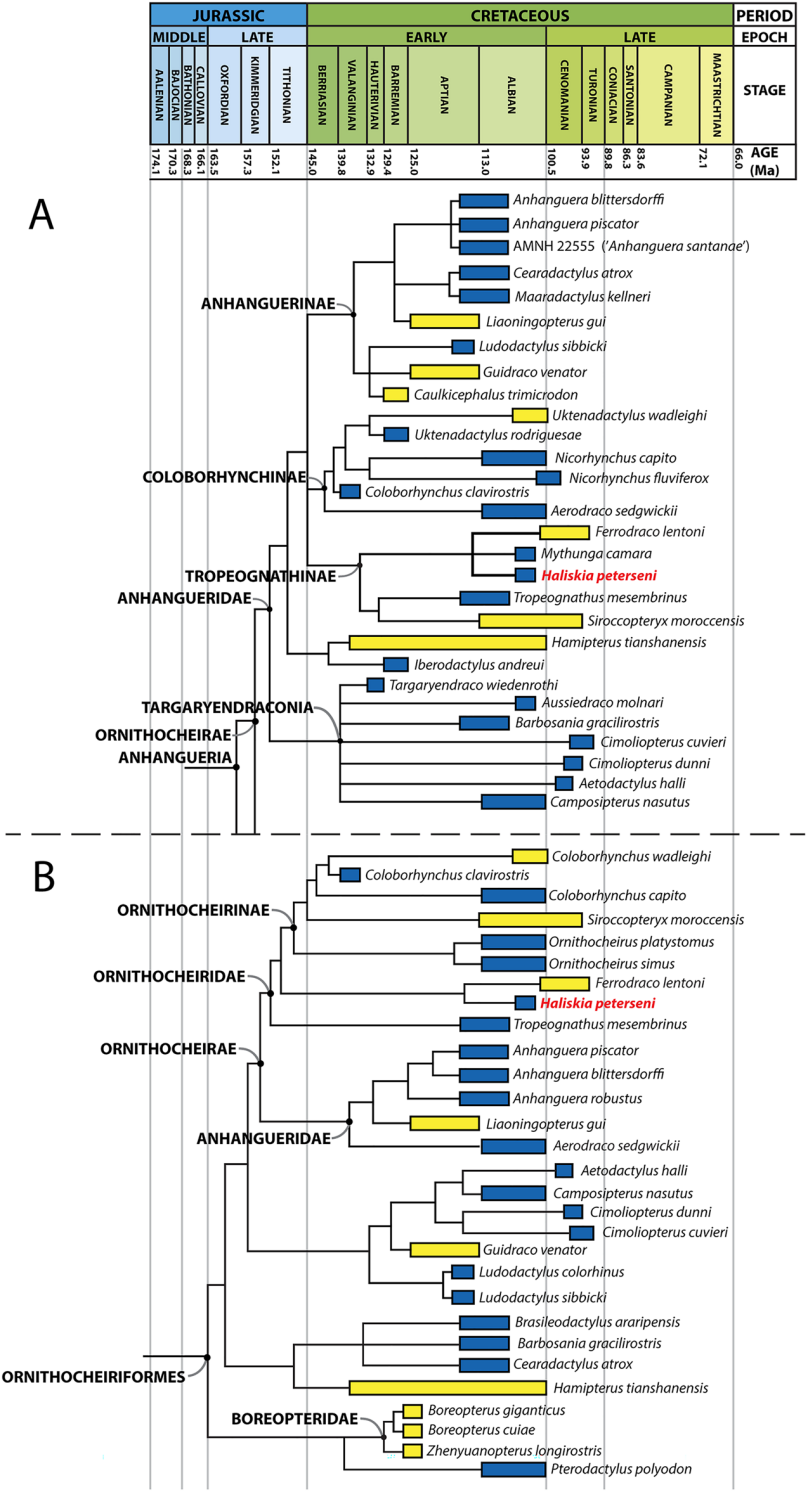
Time-calibrated strict consensus tree showing the phylogenetic relationships of *Haliskia peterseni* gen. et sp. nov. within the Anhangueria. The box next to each taxon denotes its temporal range (including stratigraphic uncertainty), colour of each box denotes the palaeoenvironmental setting (yellow = terrestrial; blue = marine). Strict consensus tree based on the matrix. (**A**) Strict consensus tree based on the matrix of Holgado and Pêgas^16^ with *Haliskia peterseni* included; and (**B**) strict consensus tree  based on the matrix of Andres^68^, with^11^,*Ferrodraco lentoni* and *Haliskia peterseni* included. Modified from Pentland, et al.^12^.

*Haliskia peterseni* possesses several anhanguerian synapomorphies, including the presence of premaxillary and mandibular crests, and a scapula that is substantially shorter than the coracoid (scapula: coracoid < 0.80)^69^. The results of the phylogenetic analyses presented here support placement of *Haliskia peterseni* within the less inclusive clade Ornithocheirae; however, its precise position within this clade is less clear. Both strict consensus trees support a close relationship between *Ferrodraco lentoni* and *Haliskia peterseni*, with the two resolved in a polytomy with *Mythunga camara* (Fig. 11A) or as sister taxa (Fig. 11B). This is not unsurprising, given that *Mythunga* was also recovered from the upper Albian Toolebuc Formation, whereas *Ferrodraco* is from the Cenomanian–lowermost Turonian Winton Formation, also from the Eromanga Basin. A close relationship between *Ferrodraco* and *Mythunga* has been supported in various phylogenetic analyses^11,12,16,23,25,26^. Moreover, these results support the interpretation of an anhanguerian radiation in the Australian Cretaceous, coinciding with the marine transgression in the Eromanga Basin, noted by Richards, et al.^23^. A similar conclusion can be drawn regarding a Gondwanan radiation of anhanguerians, albeit with less certainty, as this conclusion is dependent on the Tropeognathinae consisting of *Tropeognathus* (lower Albian; Romualdo Formation), *Siroccopteryx* (Cenomanian; Kem Kem Group), and the Australian anhanguerians.

### Wingspan estimate

Given that the humerus, ulna, and radius are missing, and that manual phalanges IV-2, IV-3 and IV-4 are incomplete, the wingspan of *Haliskia peterseni* cannot be determined with certainty. However, it can be estimated, based on comparisons with other osteologically mature anhanguerian pterosaurs. The anteroposterior lengths of the coracoid, metacarpal IV and manual phalanx IV-1 of *Haliskia* are similar to those of *Arthurdactylus conandoylei*^65^. Based on these comparisons, the wingspan of *Haliskia* is estimated at approximately 4.60 m, slightly larger than that of *Ferrodraco*^11^ and within the size range estimates of *Mythunga*^19,25^. Although previous estimates suggest the wingspan of *Thapunngaka* varied between 6 and 7 metres^23^, this is based solely on the implied length of the mandibular symphysis. Despite resolving *Thapunngaka* and *Ferrodraco* as sister taxa, Richards, et al. ^23^ did not base their wingspan estimate on the latter. Moreover, comparisons with the closely related *Tropeognathus mesembrinus* were deemed problematic, because the symphysis length in that taxon is a third of its overall skull length^49^, thus resulting in a low mandibular symphysis: wingspan ratio^23^^; p. 12^. Instead, wingspan estimates for *Thapunngaka shawi* were based on *Anhanguera piscator*^54^, on the basis that the number of alveoli present before the mandibular symphysis is similar in both taxa^23^. However, based on the strict consensus tree presented by Richards, et al.^23^, there seems little reason to compare *Thapunngaka* with *Anhanguera piscator* when more phylogenetically proximal comparators exist. The reconstructed skull of *Thapunngaka shawi* is 650 mm in length^23^^: fig. 6^ —similar to that of *Ferrodraco*^11^. Consequently, it seems likely that there was little difference in overall skull size between these two taxa, and this in turn implies that these taxa had similar wingspans. Given that wingspan estimates based solely on isolated partial crania are highly problematic^25^, the claim that *Thapunngaka shawi* represents the largest known pterosaur from Australia cannot be supported on the basis of the evidence available.

### Feeding behaviour

The rarity of pterosaur fossils that preserve direct evidence of feeding behaviour has hindered palaeoecological research on the clade (Bestwick et al.^70^). Although content fossils (remains associated with the throat and/or thoracic cavity) are not preserved in *Haliskia*, the hyoid apparatus, dentition and inflated alveolar borders provide some insight into the feeding habit of this taxon. In life, the ceratobranchials formed a Y-shaped hyoid, as in the anhanguerian *Ludodactylus*^56^, and some istiodactylids^55,71^. The elongated Y-shaped hyoid apparatus of *Liaoxipterus brachycephalus* was initially interpreted as being indicative of a projectile tongue and an insectivorous diet, based on comparisons with the Chamaeleonidae^71,72^. However, this interpretation has since been refuted, with Y-shaped hyoids more recently compared with those of extant crows (Corvidae: *Corvus*) and associated with scavenging behaviour^55^. Elongation of the ceratobranchials has been linked to strengthening of the tongue and increased mobility, thus aiding food acquisition when extruded from the oropharyngeal cavity and food transport during retraction^55^. Given that *Haliskia peterseni* demonstrates the highest ceratobranchial: mandible length ratio of any pterosaur reported to date (70%), it is likely that it possessed a strong, muscular tongue. We suggest that the tongue of this taxon might represent an evolutionary adaptation that aided in the immobilization of live, slippery prey items against the prominent palatal ridge. Although multiple lines of evidence indicate that anhanguerians were piscivorous^70^, recent dental microwear texture analysis suggests considerable overlap between carnivory and piscivory in several anhanguerian taxa, including *Anhanguera*, *Boreopterus*, and *Lonchodraco*^73^. A notable exception is *Coloborhynchus*, which demonstrates microwear texture indicative of a broader dietary range (including vertebrates other than fish), based on comparisons with extant reptiles^73^. Given that some anhanguerians are interpreted as feeding on a variety of different food sources, the morphology of the hyoid alone cannot be used to infer dietary preference.

Inflated alveolar borders, like those seen in *Haliskia*, might have provided additional resistance against lateral forces, thereby reducing tooth abrasion and material fatigue. Indeed, such an interpretation might explain why inflated alveolar borders are often restricted to the anterior end of the jaw, which is subjected to the most strain during food acquisition. By contrast, the posterior alveoli often host apicobasally shorter teeth and consequently require less reinforcement. Inflated alveolar borders have been noted in the dsungaripteroids *Dsungaripterus weii*^74^ and *Tendaguripterus recki*^75^. Given the widespread distribution of inflated alveolar borders among pterodactyloids, it seems likely that this feature has evolved independently within several taxonomic families, coinciding with dietary shifts. As such, analysis of both dental and cranial morphology is critical to understanding dietary preferences in this clade.

**Figure 12:**
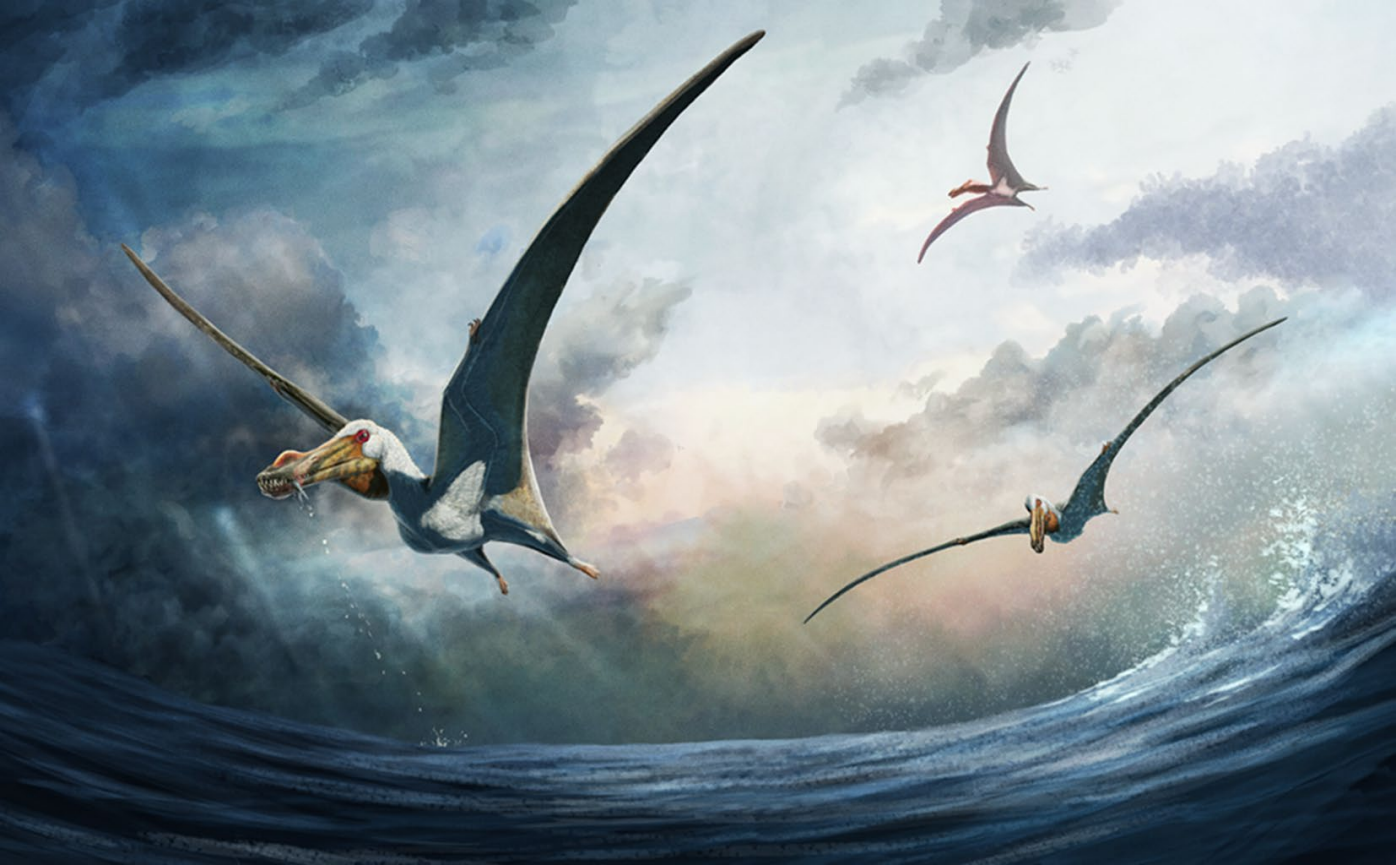
Life reconstruction of *Haliskia peterseni*. Palaeoart courtesy of Gabriel N. Ugueto, reproduced with permission.

The dental configuration of *Haliskia* is similar to that seen in other anhanguerian pterosaurs: the teeth are vertically oriented, lingually recurved, and interlock during occlusion. Thus, they form a “fish grab” dentition, preventing prey from escaping from the oropharyngeal cavity^76^ (Fig. 12). Because no replacement teeth are preserved in any of the alveoli on either side of the premaxilla, it remains unclear as to whether the first preserved premaxillary tooth of *Haliskia* represent fully functional or replacement tooth. Moreover, it is likely that *Haliskia* follows a distolingual pattern of tooth replacement, as has been observed in *Mythunga*^25^ and ‘*Coloborhynchus robustus*’ (SMNK 2302 PAL^49^), and has been suggested to be the typical mode of tooth replacement for all dentulous pterosaurs^77^. Although *Haliskia* preserves more than forty teeth, they bear little ornamentation and few possess wear facets. Comparatively few wear facets might indicate a dietary preference towards soft bodied invertebrates, as has been inferred for *Carniadactylus rosenfeldi*^78^. If this interpretation is correct, perhaps the unique combination of features present in *Haliskia* is a consequence of niche partitioning: *Haliskia* might have fed on soft-bodied invertebrates (likely cephalopods) and/or other slippery prey items, whereas the contemporaneous *Mythunga* might have targeted larger prey, as indicated by the strong striations and comparatively robust dentition.

The cosmopolitan nature of anhanguerian pterosaurs, particularly their success across Gondwana (e.g., in the Eromanga and Araripe basins), might have been enabled by niche partitioning within this clade. However, better temporal constraints will be needed at multiple localities to rigorously test this hypothesis. Additional data on this clade, as provided by *Haliskia peterseni*, sheds light on the paleoecology of anhanguerian pterosaurs, while concomitantly highlighting the taxonomic diversity of these flying reptiles in the Australian Cretaceous.

## Materials and methods

### Specimen

KK F1426 is permanently housed at Kronosaurus Korner, Richmond, Queensland, Australia. Measurements of KK F1426 were taken using digital calipers and photographed using a Nikon D750 camera and Nikon AF-S 24-85 mm lens. The specimen was mechanically prepared by K. Petersen at Kronosaurus Korner (KK), Richmond, Queensland, Australia. The bone was consolidated using Paraloid B-72 resin diluted in acetone.

Some parts of KK F1426 and the surrounding matrix were scanned using an Artec Space Spider handheld laser scanner (www.artec3d.com/portable-3d-scanners/artec-spider-v2), and the subsequent three-dimensional models were scaled and manipulated in Artec Studio 15 Professional (www.artec3d.com/3d-software/artec-studio). Figures of three-dimensional models were assembled in Adobe Photoshop 2024 and outlined and annotated in Adobe Illustrator 2024.

### Scanning using neutron tomography

KK F1426 was imaged using DINGO, the thermal-neutron tomography instrument located at the Australian Nuclear Science and Technology Organisation’s 20 MW OPAL multi-purpose reactor, Lucas Heights, New South Wales, Australia^79^. For this scan, the instrument was configured in high-flux mode, with a neutron flux-at-sample of 4.75 × 10^7^ n cm^−2^ s^−1^ (for *L*/*D* = 500), with the plate-like specimen stood vertically on a rotation stage directly in-front of a 200 × 200 × 200 mm Gd2O2S:Tb scintillation screen (RC Tritec AG), which converts neutrons to photons. The resultant photons were detected by a ZWO ASI2600MM Pro camera (liquid colled, 16-bit, 4175 × 6248 pixels) coupled with a 50 mm Carl Zeiss lens to achieve a cubic voxel size of 52.35 mm. A total of 1800 equiangular radiographic projections of 3 s each were acquired as the specimen was rotated 180° about its vertical axis, for a total scan time of 3.25 h. Data were denoised and downsampled to 104.7 mm using ImageJ 1.51 h, and the 3D reconstruction computed using Octopus Reconstruction v.8.8. KK F1426 was digitally removed from surrounding matrix using Object Research Systems Dragonfly 2022.2, and visualisation of the resultant 3D contour mesh was visualised and rendered in Blender 3.3.

Complementary CT-scans were obtained using the Imaging and Medical Beamline at the Australian Synchrotron. Neutron tomography achieved better penetration relative to the monochromatic 90 keV synchrotron-X-ray data, and better contrast and signal-to-noise relative to the 220 keV pink X-ray beam data, therefore the neutron data were used for this study.

### Taxonomic nomenclature

This study follows the taxonomy of Holgado and Pêgas^16^ and Rodrigues and Kellner^51^, both based on the phylogenetic definitions of Kellner^69^. As such, the Anhangueridae Campos and Kellner^44^ is defined as the clade including the most recent common ancestor of *Anhanguera* and *Tropeognathus* and all their descendants^69^. Following Rodrigues and Kellner^51^, the more inclusive Anhangueria includes the Anhangueridae, as well as the genera *Cearadactylus*, *Brasileodactylus*, *Ludodactylus*, and *Camposipterus*. Moreover, given the uncertain phylogenetic position of *Ornithocheirus simus*, the present work follows Rodrigues and Kellner^51^ and restricts the Ornithocheiridae to the holotype specimen.

### Phylogenetic analysis

In order to determine the phylogenetic position of KK F1426, the specimen was scored for two datasets: the data matrices of Andres^68^ and Holgado and Pêgas^16^. The Andres^68^ dataset was modified with the inclusion of the Australian pterosaurs *Mythunga camara*, *Ferrodraco lentoni,* as well as the addition of *Aussiedraco molnari*, *Thapunngaka shawi* and the present specimen. The Holgado and Pêgas^16^ matrix was modified as in Pentland, et al.^12^, through revision of scores for *Mythunga camara*, *Ferrodraco lentoni* and *Aussiedraco molnari*, and the inclusion of *Thapunngaka shawi* and KK F1426. *Thapunngaka* was placed within Anhangueria using the Holgado and Pêgas^16^ dataset, but it was found to be unstable and subsequently excluded.

After modifications, the Holgado and Pêgas^16^ dataset comprises 179 discrete characters and 75 taxa, whereas the Andres^68^ matrix comprises 275 characters (90 continuous characters, 185 discrete characters) and 182 taxa.

Phylogenetic analyses were run in TNT v.1.5 (Goloboff et al.^80^); the Holgado and Pêgas^16^ dataset was analysed following the methodology of Pêgas, et al.^27^, whereas the Andres^68^ matrix was analysed following the methodologies of those authors. All characters were weighted equally, with no additive characters considered.

### Nomenclatural acts

The electronic version of this article in Portable Document Format (PDF) will represent a published work according to the International Commission on Zoological Nomenclature (ICZN), and hence the new names contained in the electronic version are effectively published under that Code from the electronic edition alone. This published work and the nomenclatural acts it contains have been registered in ZooBank, the online registration system for the ICZN. The ZooBank LSIDs (Life Science Identifiers) can be resolved, and the associated information viewed through any standard web browser by appending the LSID to the prefix http://zoobank.org/. The LSID for this publication is: urn:lsid:zoobank.org:act:87D0EBA7-024E-4D62-B5E3-EC7814D9B7BC (*Haliskia*), and urn:lsid:zoobank.org:act:6AC664D4-AF22-4E69-95F2-713624D04B0D (*Haliskia peterseni*). The online version of this work is archived and available from the following digital repositories: PeerJ, PubMed Central and CLOCKSS.

### Supplementary Information

Supplementary Information 1.

Supplementary Information 2.

Supplementary Information 3.

## Data Availability

The datasets used and/or analysed during the current study available from the corresponding author on reasonable request.

## Abbreviations

AAOD: Australian Age of Dinosaurs Museum of Natural History, Winton, Queensland, Australia
AODF: Australian Age of Dinosaurs Fossil
AODL: Australian Age of Dinosaurs Locality
AMNH: American Museum of Natural History, New York, U.S.A
BSP: Bayerische Staatsammlung für Paläontologie und Historische Geologie, Munich, Germany
CAMSM: Sedgwick Museum, Cambridge, U.K
IWCMS: Isle of Wight County Museum Service at Dinosaur Isle, Sandown, Isle of Wight, U.K
KK: Kronosaurus Korner, Richmond, Queensland, Australia
KK F: Kronosaurus Korner Fossil
LINHM: Long Island Natural History Museum, New York City, U.S.A
QM: Queensland Museum, Brisbane Australia
QM F: Queensland Museum Fossil, Brisbane, Australia
