# Supplementary material for: Haliskia peterseni, a new anhanguerian pterosaur from the late Early Cretaceous of Australia
Source: Sci Rep. 2024 Jun 12;14:11789. doi: 10.1038/s41598-024-60889-8 (PMC11169243; doi:10.1038/s41598-024-60889-8)

*Haliskia peterseni*, a new anhanguerian pterosaur from the late Early Cretaceous of Australia

Adele H. Pentland^1,2^, Stephen F. Poropat^1,2,3^, Ruairidh J. Duncan^3,4^, Alexander W.A. Kellner^5^, Renan A. M. Bantim^6^, Joseph J. Bevitt^7^, Alan M. Tait^4^, Kliti Grice^1^

^1^ WA-Organic and Isotope Geochemistry Centre, School of Earth and Planetary Sciences, Curtin University, Perth, Western Australia, Australia

^2^ Australian Age of Dinosaurs Natural History Museum, The Jump-Up, Winton, Queensland 4735, Australia

^3^ School of Biological Sciences, Monash University, Clayton, Victoria 3800, Australia

^4^ Museums Victoria, PO Box 666, Melbourne, Victoria 3001, Australia

^5^ Laboratório de Sistemática e Tafonomia de Vertebrados Fósseis, Setor de Paleovertebrados, Departamento de Geologia e Paleontologia, Museu Nacional, Universidade Federal do Rio de Janeiro, Rio de Janeiro, Brazil

^6^ Museu de Paleontologia Plácido Cidade Nuvens, Universidade Regional do Cariri, Santana do Cariri, CE, Brazil

^7^ Australian Centre for Neutron Scattering, Australian Nuclear Science and Technology Organisation, Sydney, Australia

Corresponding Author:

Adele H. Pentland^1^

10268 Winton Eskdale Road, Corfield, Queensland, 4733, Australia

Email address: pentlandadele@gmail.com

Supplementary information A

**Table S1.** Measurements of the alveolar diameters observed in *Haliskia peterseni* (KK F1426) in millimetres. Measurements based on incomplete alveoli are indicated with an asterisk (*).

| **Element** | **Alveolus** | **Mesiodistal length**  **(mm)** | **Labiolingual width**  **(mm)** | **Observations** |
| --- | --- | --- | --- | --- |
| **Left premaxilla–**  **maxilla** | 1 | 5 | 7 | Alveolus occupied by tooth |
|  | 2 | 10 | 4 |  |
|  | 3 | 10 | 5* |  |
|  | 4 | 6 | 5* |  |
|  | 5 | 6 | 5* |  |
|  | 6 | 9 | 8 |  |
|  | 7 | 9 | 9 |  |
|  | 8 | 10 | 8 | Posterior margin of alveolus incompletely preserved |

**Table S2.** Interalveolar spacing of *Haliskia peterseni* (KK F1426) in millimetres. Measurements taken from outer rim to outer rim. Measurements based on incomplete alveoli are indicated with an asterisk (*).

| **Left premaxilla–**  **maxilla** | **Mesiodistal length of interalveolar**  **space (mm)** |
| --- | --- |
| **1–2** | 2 |
| **2–3** | 1* |
| **3–4** | 2 |
| **4–5** | 2 |
| **5–6** | 4 |
| **6–7** | 7 |
| **7–8** | 13 |

**Fig. S1:** Various isolated teeth of *Haliskia peterseni* gen. et sp. nov., (A) isolated tooth, (B) isolated tooth, (C) two isolated teeth and first manual phalanx of digit I, (D) isolated tooth, (E) isolated tooth, (F) isolated tooth. Abbreviations: mdI.1, first manual phalanx of digit I. Scale bar = 20 mm.


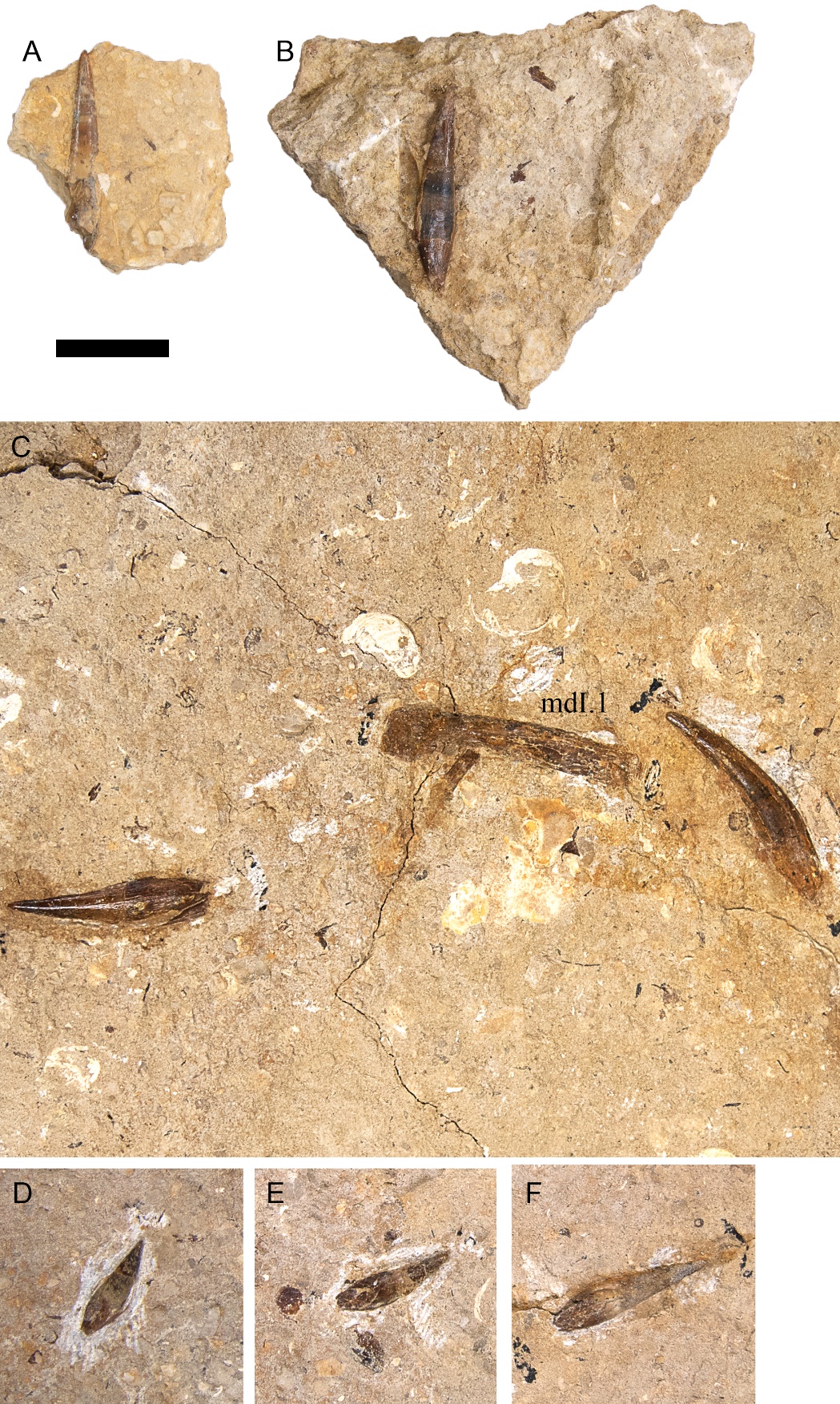


**Fig. S2:** Three-dimensional surface render of dorsal ribs, left scapulocoracoid, left and right manual phalanx IV-2, left femur, left tibia, pedal phalanges and metatarsal of *Haliskia peterseni* gen. et sp. nov. Abbreviations: cor, coracoid; fe, femur; gas, gastralia, l, left; mt, metatarsal; ph, phalanx; pphd, pedal phalanx; r, right; ri, rib; sca, scapula; t, tibia. Scale bar = 50 mm.


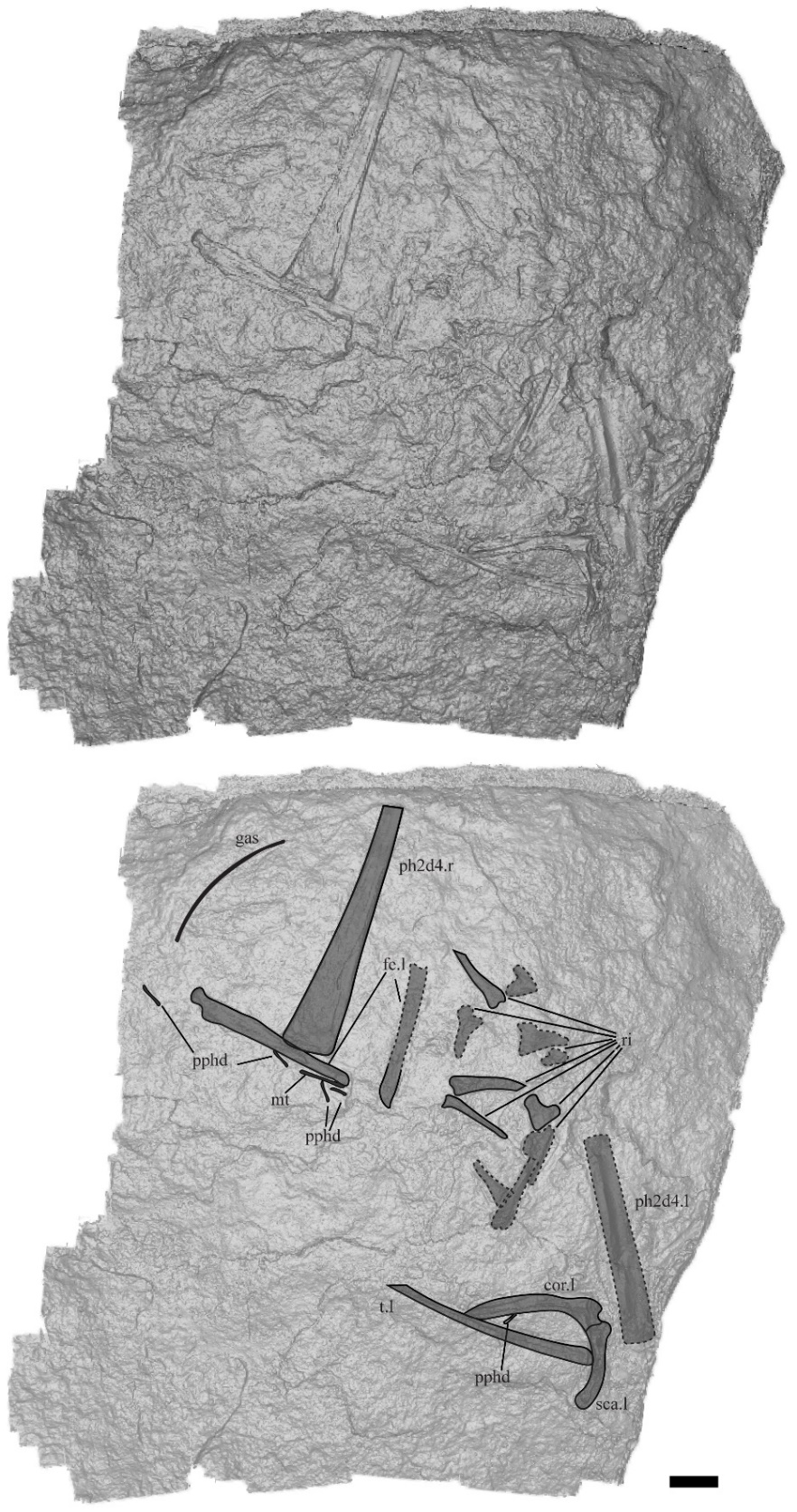

Supplement: Supplementary file 2 — Supplementary Information 2. [file 41598_2024_60889_MOESM2_ESM.docx]
